# Metabolic characterization of the natural progression of chronic hepatitis B

**DOI:** 10.1186/s13073-016-0318-8

**Published:** 2016-06-10

**Authors:** Johannes C. Schoeman, Jun Hou, Amy C. Harms, Rob J. Vreeken, Ruud Berger, Thomas Hankemeier, Andre Boonstra

**Affiliations:** Department of Analytical Biosciences, Leiden Academic Center for Drug Research, Leiden University, Einsteinweg 55, 2333 CC Leiden, The Netherlands; Netherlands Metabolomics Centre, Leiden University, Einsteinweg 55, 2333 CC Leiden, The Netherlands; Department of Gastroenterology and Hepatology, Erasmus MC—University Medical Center Rotterdam, Wytemaweg 80, Room Na-1011, 3015 CE Rotterdam, The Netherlands; Present address: Discovery Sciences, Janssen R&D, Turnhoutseweg 30, 2340 Beerse, Belgium

**Keywords:** Metabolomics, Chronic hepatitis B, Liver, Viral hijacking, Clinical phases

## Abstract

**Background:**

Worldwide, over 350 million people are chronically infected with the hepatitis B virus (HBV) and are at increased risk of developing progressive liver diseases. The confinement of HBV replication to the liver, which also acts as the central hub for metabolic and nutritional regulation, emphasizes the interlinked nature of host metabolism and the disease. Still, the metabolic processes operational during the distinct clinical phases of a chronic HBV infection—immune tolerant, immune active, inactive carrier, and HBeAg-negative hepatitis phases—remains unexplored.

**Methods:**

To investigate this, we conducted a targeted metabolomics approach on serum to determine the metabolic progression over the clinical phases of chronic HBV infection, using patient samples grouped based on their HBV DNA, alanine aminotransferase, and HBeAg serum levels.

**Results:**

Our data illustrate the strength of metabolomics to provide insight into the metabolic dysregulation experienced during chronic HBV. The immune tolerant phase is characterized by the speculated viral hijacking of the glycerol-3-phosphate–NADH shuttle, explaining the reduced glycerophospholipid and increased plasmalogen species, indicating a strong link to HBV replication. The persisting impairment of the choline glycerophospholipids, even during the inactive carrier phase with minimal HBV activity, alludes to possible metabolic imprinting effects. The progression of chronic HBV is associated with increased concentrations of very long chain triglycerides together with citrulline and ornithine, reflective of a dysregulated urea cycle peaking in the HBV envelope antigen-negative phase.

**Conclusions:**

The work presented here will aid in future studies to (i) validate and understand the implication of these metabolic changes using a thorough systems biology approach, (ii) monitor and predict disease severity, as well as (iii) determine the therapeutic value of the glycerol-3-phosphate–NADH shuttle.

**Electronic supplementary material:**

The online version of this article (doi:10.1186/s13073-016-0318-8) contains supplementary material, which is available to authorized users.

## Background

Worldwide, over 350 million people are chronically infected with the hepatitis B virus (HBV) and are at increased risk of developing progressive liver diseases, including fibrosis, liver failure, or hepatocellular carcinoma (HCC), over the course of several decades [1, 2]. Chronic HBV infection can be divided into four progressively distinct clinical phases based upon serum levels of HBV DNA, alanine aminotransferase (ALT), and HBV envelope antigen (HBeAg). These four phases are the immune tolerant (IT), immune active (IA), inactive carrier (IC), and HBeAg-negative (ENEG) phases. The increased ALT levels in the IA and ENEG phases reflect hepatic injury due to viral activity and immune activity. Even though these phases are used in clinical practice for deciding on therapeutic interventions [3], not much is known about the underlying metabolic mechanisms associated with each and/or the progressive nature of the disease. HBV replicates in the liver, which also acts as the central hub for metabolic and nutritional regulation, emphasizing the interlinked nature of the metabolism and the disease. Hence, a clear metabolic understanding of the progressive driving forces and diverse clinical outcomes is important in the management of chronic hepatitis B—for instance, to provide prediction markers for disease progression and candidate targets for therapeutic intervention. In vitro systems are not reliable models for chronic HBV infection since they do not represent the persistence, complexity, and progressive nature of the disease. Animal models do not appropriately mimic the pathogenic and immunological responses experienced during human HBV infection, hindering effective translational studies [4, 5]. Thus, the use of material obtained from patients is crucial to study and understand chronic HBV infection.

Metabolomics is an established, continuously improving tool used to study the metabolome through targeted or untargeted approaches, providing static observational and/or longitudinal readouts from a dynamic system [6–9]. Targeted metabolomics, which focuses on a predefined biology-driven subset of metabolite classes, is ideal for low concentration metabolites, whereas untargeted metabolomics is more suitable for measuring high concentration metabolites [10]. Metabolomics data aid in identifying and understanding the metabolic perturbations leading to dysregulated homeostasis and stress, e.g., during viral infection [11–14]. Subsequently, the contribution of these metabolic perturbations to the pathogenicity of the virus can be determined. Several metabolomics studies have been done to assess the metabolic host–pathogen interactions during chronic HBV infection. Targeted metabolomics approaches have identified the phospholipid [15, 16], triglyceride [15, 17, 18], sphingomyelin [15, 19], and free fatty acid [16, 20] metabolic pathways as affected during chronic HBV infection. Untargeted approaches have also been used to investigate metabolic changes experienced during chronic HBV infection [21–23] and have primarily focused on HBV-related cirrhosis and HCC [15, 24–26].

Here we present the first discovery metabolomics study of chronic HBV infection, shedding light on the progressive metabolic alterations over the different clinical phases. We used targeted metabolomics platforms to illuminate the phospholipid, triglyceride, sphingomyelin, amino acid, acyl-carnitine, and signaling lipid profiles to characterize the progressive nature of chronic HBV infection. We identified the most profound changes to occur in choline glycerophospholipids, plasmalogens, very long chain triglyceride species, and urea cycle intermediates across the progression of the four clinical phases of chronic HBV infection. Our data support the viral hijacking and persistent impairment of the glycerol-3-phosphate–NADH shuttle, modulating the host lipid profile. These events are central to understanding the metabolic perturbations reflective of the natural progression of chronic HBV infection.

## Methods

### Study population

Sixty-nine chronic HBV patients and 19 healthy controls (HCs) were included in this discovery study. Blood was prospectively collected in SST tubes at the Erasmus MC and centrifuged to separate the serum and stored at −80 °C until analysis. Patients were treatment naïve and excluded if they had other chronic infections (HIV, hepatitis C virus) or a body mass index of 31 or more. This study was conducted in accordance with the guidelines of the Declaration of Helsinki and the principles of Good Clinical Practice. The ethical review board of the Erasmus MC approved the study and informed consent was obtained from all patients who were asked to donate blood.

### Definition of chronic HBV clinical phases

Serum ALT was measured on an automated analyzer, qualitative serum HBV surface antigen (HBsAg), HBeAg, and anti-HBeAg antibodies were measured on an Architect Abbott analyzer, and serum HBV DNA levels were measured using the COBAS AmpliPrep-COBAS Taq-Man HBVv2test (CAP-CTM; Roche Molecular Systems, Indianapolis, IN, USA). Based on serum HBV DNA, ALT levels, and HBeAg presence at the time of sampling, patients were categorized into four clinical HBV phases according to international guidelines [27]. IT patients (*n* = 18) had detectable serum HBeAg and repetitive normal ALT values (<40 U/L). IA patients (*n* = 12) and ENEG patients (*n* = 19) had repetitive or intermittent abnormal serum ALT (>40 U/L) values and HBV DNA levels >2000 IU/mL. IC patients (*n* = 20) were HBeAg-negative and had both repetitive normal ALT values (<40 IU/L) and HBV DNA levels below 20,000 IU/mL.

### Targeted liquid chromatography–mass spectrometry metabolomics

Targeted metabolomics analyses were done using standard operating procedures from previously published methods. Detailed procedures and target lists are provided in the Additional file 1: Methods and a brief overview of the five platforms used is given in Table 1.

**Table 1:** Metabolomics platforms: volumes, sample preparation, and analytical instruments

Targeted metabolomics platform	Volume of serum used (μL)	Sample prep method	Analytical platform	Metabolite class coverage	Platform targets (n)		
					Total	Quality control passed	Percentage of missing data
Biogenic amine [51]	5	Protein precipitation and AccQTag derivatization	UPLC-Xevo-TQMS	Amino acids, catecholamines and polyamines	100	38	0.03
Positive lipid [52]	10	Isopropyl alcohol extraction	UPLC-QToF	Phospholipids, cholesterol esters, di/triglycerides and sphingomyelins	250	140	0.11
Negative lipid [52]	20	Methanol extraction	UPLC-QToF	Free fatty acids and phospholipids	150	59	0.17
Oxylipins [53]	250	Oasis HLB solid-phase extraction	HPLC–QqQ-MS	Hydroxylated fatty acids, prostaglandins, and thromboxanes	120	35	0.07
Acyl-carnitines	10	Protein precipitation	UPLC- Xevo-TQMS	Acylcarnitines, TMAO, choline, betaine	50	26	0

*QqQ-MS* triple quadrupole mass spectrometry, *QToF* quadrupole time-of-flight, *TQMS* triple quadrupole mass spectrometry, *TMAO* trimethylamine-N-oxide, *UPLC* ultra performance liquid chromatography

Quality control (QC) samples consisted of equally pooled volumes of all study samples. A set of QC samples was then included during the analyses of the experimental groups on the individual metabolomic platforms and evenly distributed through the randomized samples prior to liquid chromatography–mass spectrometry (LC–MS) analyses. In addition, independent duplicate samples (10–15 %) were randomly selected where sample volume allowed.

After LC–MS analyses, peak integration was done using the instrument’s software and the relative ratios between metabolites and their corresponding internal standards were determined. Using the QC samples and duplicate samples, a double QC approach was applied to include metabolites that were reliably measured by the individual metabolomics platforms by reporting and using only those metabolites for which both duplicate samples and QC samples showed a relative standard deviation <30 %. After QC, a data set comprising 88 cases and 314 metabolites was constructed, with all missing data being replaced by half the minimal intensity value of the corresponding metabolite.

### Statistical data analyses

SPSS 21.0 (SPSS Inc., Chicago, IL, USA) was used for Fisher’s exact tests on the patient cohort characteristics presented as frequencies and ANOVA on the continuous values. A combination of univariate and multivariate bioinformatics approaches was performed using the R script-based online tool Metaboanalyst 3.0, a comprehensive tool suitable for analyzing metabolomics data [28, 29]. The metabolomics data sets were log transformed and auto-scaled prior to bioinformatics analyses. For the analyses between HCs and IT patients, significant metabolites were identified per metabolomics platform based on the following criteria: (i) a *p* value <0.05 using the independent student *t*-test; and (ii) a fold change ≥1.20 or ≤0.80, indicating a 20 % increase or decrease. The false discovery rate’s *q* values are reported for every reported *p* value. Multivariate principal component analysis was also done to visualize the natural distribution of the data.

The multivariate ANOVA test was used to identify the significant changes (*p* < 0.05) across the four clinical phases of chronic HBV infection in conjunction with K-means clustering. The K-means clustering was performed in MATLAB using *kmeans* and “correlation” as the distance measure on the metabolites in order to examine changes in their levels during the course of chronic HBV infection, using all 314 metabolites and 88 patients. Its algorithm partitions the metabolites into K mutually exclusive clusters (k is the number of desired clusters), taking into account the measurement of each metabolite under multiple conditions, in this case HCs plus all four clinical phases. Within each cluster, metabolite measurement patterns are as close to each other as possible, while as far away from those in other clusters as possible. The partition was repeated with metabolites reassigned among clusters at each iteration until the sum of point-to-centroid distance reached a minimum [30]. The pattern analysis was performed sequentially with k assigned with 12, 16, 20, and 24 each time.

## Results

### Baseline characteristics of the study population

**Fig. 1:**
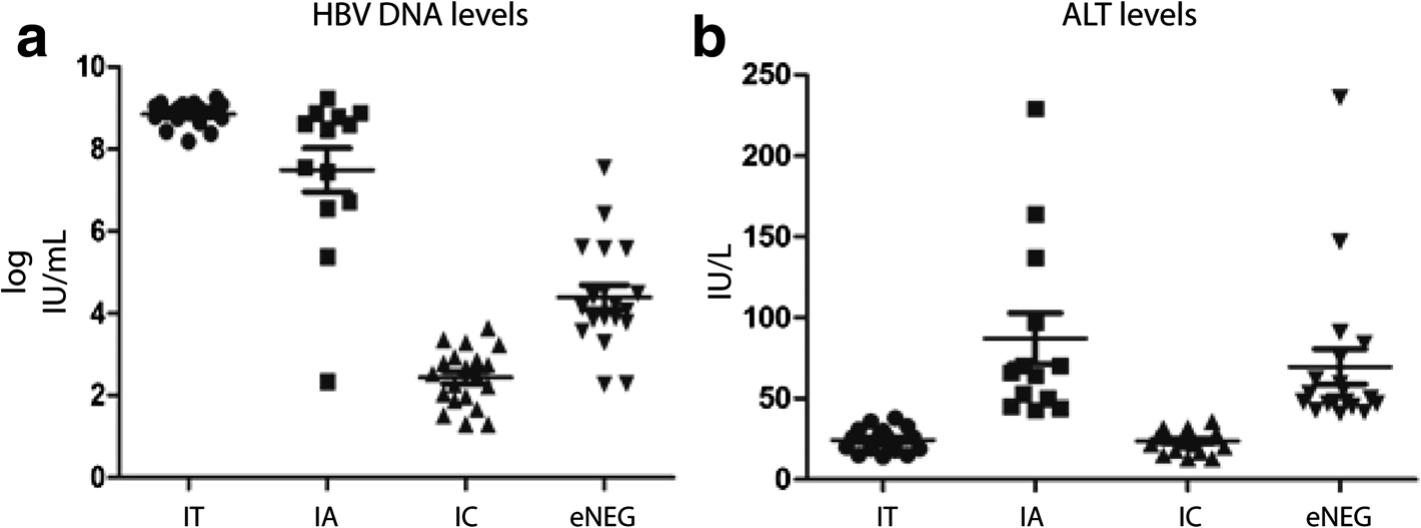
Clinical parameters. Baseline serum characteristics of patients with chronic HBV infection divided into four clinical phases based on ALT and HBV DNA levels. Serum samples of 69 chronic HBV patients were measured for their levels of **a** HBV DNA and **b** ALT levels

For the metabolomics characterization of chronic HBV infection, a cohort of treatment-naïve patients was chosen and patients with other comorbidities and/or advanced liver fibrosis were excluded from the study. Due to the progressive nature of chronic HBV infection over time, IT and IA patients are younger than IC and ENEG patients (*p* = 0.03; Table 2). The asymptomatic IT and IC groups have a larger female representation due to routine HBsAg testing during pregnancy and subsequent referrals. This gender ratio is reversed in the IA and ENEG groups (Table 2). The ratios of HBV genotypes are evenly spread in the cohort, except for the IT and ENEG groups where genotypes B/C and D are dominant, respectively (Table 2). Owing to the stringent definition criteria, differences in ALT (Fig. 1a) and HBV DNA (Fig. 1b) levels are observed between the clinical phases.

**Table 2:** Baseline characteristics of the patient cohort

Characteristics	Total cohort	Healthy control (HC)	Immune tolerant (IT)	HBeAg-positive active hepatitis (IA)	Inactive carrier (IC)	HBeAg-negative active hepatitis (ENEG)
Number of patients	88	19	18	12	20	19
Demography						
Age, years (SE)	35.26 (11.58)	-	30.89 (1.77)	31.75 (2.60)	39.85 (3.02)	37.68 (2.75)
Gender, n**						
Female	41	7 (17.1 %)	13 (31.7 %)	5 (12.2 %)	14 (34.2 %)	2 (4.9 %)
Male	41	6 (14.6 %)	5 (12.2 %)	7 (17.1 %)	6 (14.6 %)	17 (41.5 %)
		7 missing				
BMI, kg/m^2^ (SE)	24.64 (6.40)	-	25.93 (3.27)	23.08 (1.12)	24.41 (0.70)	24.66 (0.79)
Race, n*						
Asian	44 (63.77 %)	-	16 (36.4 %)	9 (22.7 %)	8 (18.21 %)	10 (22.7 %)
Caucasian	9 (13.04 %)	-	0 (0.0 %)	3 (33.3 %)	2 (22.2 %)	4 (44.4 %)
African	8 (11.59 %)	-	0 (0.0 %)	0 (0.0 %)	5 (62.5 %)	3 (37.5 %)
Other	8 (11.59 %)	-	2 (25.0 %)	0 (0.0 %)	4 (50 %)	2 (25.0 %)
Virology						
Log HBV DNA, IU/ml (SE)***	5.55 (2.82)	-	8.84 (0.07)	7.40 (0.57)	2.43 (0.15)	4.39 (0.30)
HBV genotype A/B/C/D**	13/16/20/20	-	0/8/8/2	3/2/6/1	6/5/2/6	4/1/3/11
Chemistry/hematology						
ALT, IU/l (SE)***	48.17 (43.56)	-	24.50 (1.72)	88.83 (16.93)	23.90 (1.43)	69.53 (10.91)

*BMI* body mass index, *SE* standard error

Fisher’s exact *p* value: *p < 0.05, **p < 0.01

***ANOVA *p* value < 0.01

### Metabolic characterization of the IT phase of chronic HBV infection versus the HCs

**Fig. 2:**
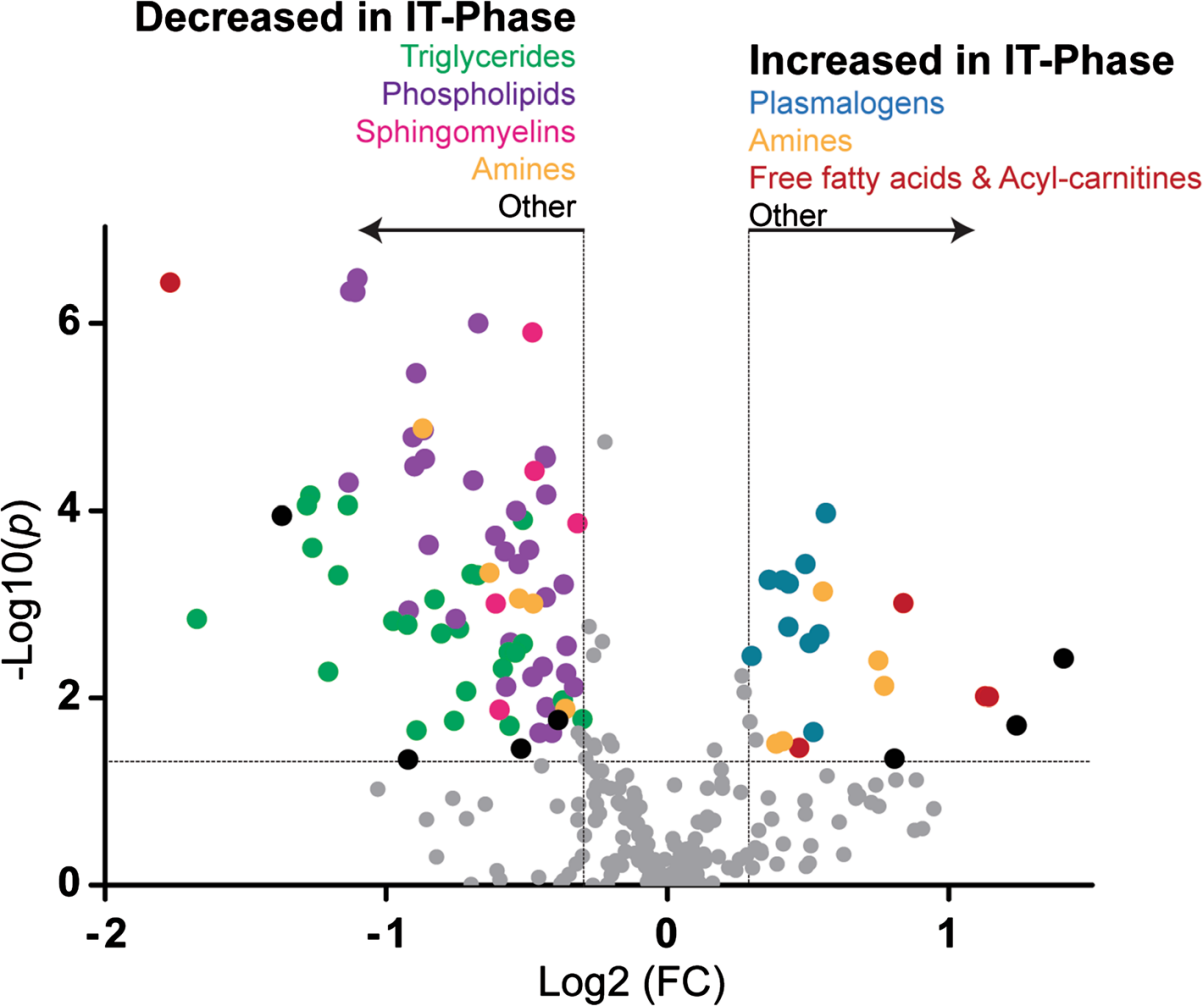
Volcano plot of the significant serum metabolites between the HCs and the IT phase. Metabolites with a fold change (*FC*; *x-axis*) threshold of ≥1.20 or ≤0.80 and *t*-tests (*y-axis*) threshold of 0.05 are identified as significant and represented by colored circles ordered by the metabolite class. The further its position away from (0,0), the more significant the metabolite. Metabolite classes are as follows: triglycerides, *green*; phospholipids, *purple*; sphingomyelins, *pink*; amines, *orange*; plasmalogens, *blue*; free fatty acids and acyl carnitines, maroon; others, *black*

To investigate and understand the metabolic alterations during the progressive clinical phases of a chronic HBV infection, we first characterized HCs versus the IT phase, representing the onset of chronic HBV. Multivariate and univariate data analyses were performed to identify metabolic pathways altered in the IT phase. The principal component analysis showed natural clustering with partial overlap observed between the HC and IT sample groups (Additional file 2: Figure S1). For univariate analysis, fold change in combination with *t*-tests was used and the identified significant metabolites visualized in a volcano plot (Fig. 2; Additional file 2: Table S1). The volcano plot highlighted 92 significant metabolites belonging to the phospholipid, plasmalogen, triglyceride, amino acid, sphingomyelin, free fatty acid, and acyl-carnitine metabolic pathways listed in Table 3. Under closer inspection, the univariate/supervised analysis reveals primarily uniform class responses, indicating definite metabolic rearrangement. The down-regulated metabolites are dominated by triglycerides, phospholipids, lysophospholipids, and sphingomyelins in the IT phase compared with HCs. In contrast, plasmalogens (specialised vinyl ether-linked phospholipids), free fatty acids, and acyl-carnitines had elevated levels in the IT phase compared with HCs. Clinically, the IT phase is characterized by high levels of HBV replication and negligible hepatic injury; hence, the dysregulated lipid profile observed is most likely due to metabolic hijacking occurring during the HBV life cycle.

**Table 3:** Significant serum metabolites identified between HCs and the IT phase

Targeted metabolomics platform	Class	Metabolite species identified as significant between the HCs and IT phase	Trend in the IT phase
**Phospholipids**			
Positive lipids	Phosphatidylcholines (PC)	C32:1, C32:2, C34:1, C34:3, C34:4, C36:3, C36:4, C36:6, C38:3	Decreased
	Phosphatidylethanolamines (PE)	C38:4	Decreased
**Plasmalogens**			
Positive lipids	Plasmalogen phosphatidylcholines (pPC)	C34:2, C36:2, C36:3, C38:6	Increased
	Plasmalogen phosphatidylethanolamines (PE)	C38:5, C38:7^#^	Increased
**Lysophospholipids**			
Negative lipids	Lysophosphatidylcholines (LPC)	*sn1*: C14:0, C15:0, C16:1, C18:1, C18:2, C18:3 (ω3ω6), C20:3 (ω3ω6), C20:3 (ω9), C20:4, C22:4, C22:5 (ω6)	Decreased
		*sn2*: C14:0, C16:1, C18:1, C18:2, C18:3 (ω3ω6), C20:3 (ω3ω6), C20:4	
	Lysophosphatidylethanolamines (LPE)	C18:0, C18:1, C18:2^#^, C20:3 (ω3ω6), C20:4, C22:5^#^ (ω3)	Decreased
**Plasmalogen lysophospholipids**			
Negative lipids	Plasmalogen lysophosphatidylcholines (pLPC)	C16:0, C16:1, C18:1, C18:2	Increased
**Triglycerides**			
Positive lipids	Triglycerides	C42:1, C44:2, C46:1, C46:2, C46:3, C48:1, C48:2, C48:3, C48:4^#^, C50:0, C50:1, C50:2, C50:3, C50:4, C50:5, C51:1, C51:2, C51:3, C52:1, C52:2, C54:1, C54:2, C55:2, C56:5, C58:5	Decreased
**Amines**			
Biogenic amines	Amino acids	Tryptophan^#^	Decreased
	Glutathione	Glutathione	Decreased
	Dipeptides	γ-Glutamylglutamine	Decreased
	Amino acids metabolites	Kynurenine, saccharopine	Decreased
	Hepatic injury	4-Hydroxyproline, pipecolic acid	Increased
	Glycine metabolism	Betaine^#^, sarcosine^#^	Increased
	Other	s-Methylcysteine	Increased
**Sphingomyelins**			
Positive lipids	Sphingomyelins (SM)	C18:1/14:0, C18:1/21:0, C18:1/23:0, C18:1/25:0, C18:1/25:1	Decreased
**Free fatty acids and Acyl-carnitines**			
Negative lipids	Free fatty acids (FFA)	C18:2, C20:2	Increased
Acyl-carnitines	Acyl-carnitines	Oleylcarnitine (C18:1)^#^, Linoleylcarnitine (C18:2)	Increased
		Nonanoylcarnitine (C9:0)	Decreased
**Others**			
Positive lipids	Cholestrol esters (CE)	C18:3	Decreased
Oxylipins	Oxylipins	11-HDoHE^#^, 14-HDoHE^#^, 10-HDoHE^#^	Increased
		5-HETrE, 8,9-DiHETre^,#^, 12,13-DiHOME^,#^	Decreased

The metabolites identified as significant based on *t*-test and fold change are divided into their different classes together with their trend. All metabolites had a *q* value <0.05 unless otherwise indicated

^#^ *q* value >0.05

### Metabolomics characterization of the natural course of a chronic HBV infection

**Fig. 3:**
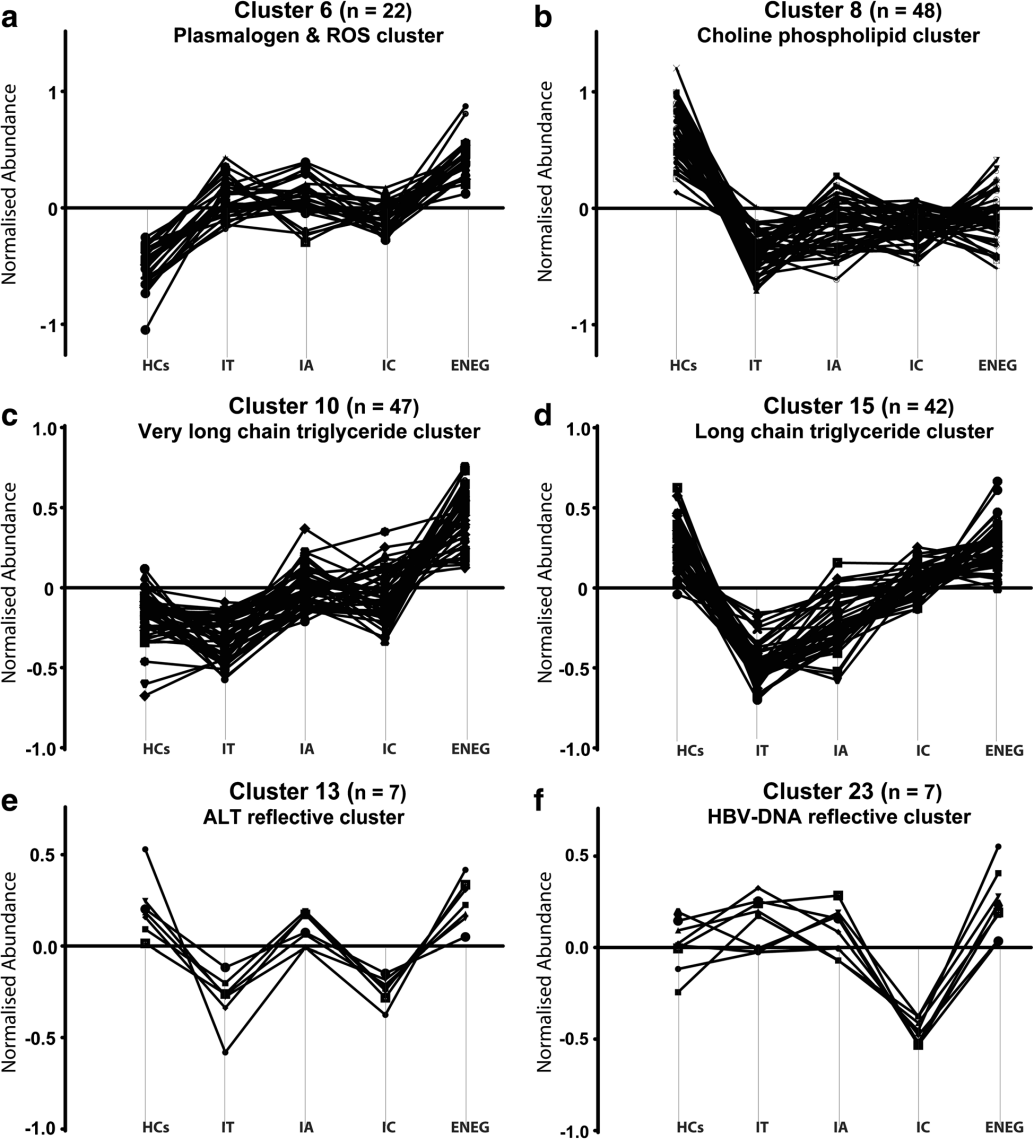
Serum metabolite pattern analyses. **a** Cluster 6, the plasmalogen and reactive oxygen species (*ROS*) cluster, showed a “stable” elevated trend against the HCs during the progression of the four clinical phases. **b** Cluster 8, the choline phospholipid cluster, showed a “stable” reduced trend over the four clinical phases against the HCs. **c** Cluster 10, the very long chain triglyceride cluster, represents metabolites with subtle changes at the start of chronic HBV infection but increased significantly over the progression of the four clinical phases. **d** Cluster 15, the long chain triglyceride cluster, grouped metabolites with a significant reduction between the HCs and the IT phase, after which their levels increased again as the disease progressed. **e** Cluster 13, the ALT reflective cluster, grouped metabolites following the same trend as ALT with increased levels in the IA and ENEG phases. **f** Cluster 23, the HBV DNA cluster, represents metabolites reflective of HBV DNA levels, which are lowest during the IC phase. The *y-axis* is the abundance of metabolites normalized to the overall mean of the metabolites across all samples

Given the interdependent nature of metabolic pathways, functionally related metabolites might have similar response patterns. To examine this, a K-means pattern analysis was performed on the total metabolomics data set comprising 88 cases and 314 metabolites. A pattern of 24 clusters was selected as the most distinct pattern describing the metabolomics data (Additional file 2: Figure S2; Additional file 3: Table S2). Subsequently, 6 of the 24 clusters were chosen for further study based on the profile and the number of metabolites (n > 5) clustering within each and the correlation between profiles and fluctuation of ALT and HBV DNA levels (Fig. 3). Pattern analysis identifies similarly responding metabolites but is not a measure of significance and, therefore, an ANOVA approach was used between the four clinical phases to identify significantly changing metabolites (Table 4; Additional file 2: Table S3).

**Table 4:** Serum metabolites identified as changing significantly over the progression of chronic HBV infection

Targeted metabolomics platform	Metabolite class	Metabolite species identified as changing significantly during the progression of chronic HBV
**Cluster 6: stable increased**		
Biogenic amines	Amines	Carnitine, ornithine
**Cluster 8: stable decreased**		
Acyl-carnitines	Acylcarnitines	Nonaylcarnitine
Negative lipids	Lysophosphatidylcholine (LPC)	*sn1*, C20:3 (ω3ω6), *sn2*, C20:3 (ω3ω6)
**Cluster 10: increasing over the four phases**		
Acyl-carnitines	Acyl-carnitines	Isovalerylcarnitine, 2-methylbutyroylcarnitine, stearoylcarnitine
Biogenic amines	Amino acids	Phenylalanine, glutamic acid, methionine
	Amines	Choline
Positive lipids	Diacylglycerol (DG)	C36:2
	Triglycerides (TG)	C54:2, C54:3, C54:4, C55:3, C56:3, C56:4, C56:5, C56:6, C58:5
Negative lipids	Free fatty acids	C20:4 (ω6)
**Cluster 13: reflective of ALT levels**		
Biogenic amines	Amino acid metabolite	Kynurenine
**Cluster 15: increasing over the four phases**		
Positive lipids	Phosphatidylcholine (PC)	C38:4
	Sphingomeylins (SM)	C18:1/25:0
	Triglycerides (TG)	C42:1, C52:2, C55:2
Negative lipids	Lysophosphatidylethanolamine (LPE)	C18:1, C20:3 (ω3ω6), C20:4
**Cluster 23: reflective of HBV DNA levels**		
Acyl-carnitines	Acylcarnitines	Decanoylcarnitine
Negative lipids	Free fatty acids	C20:3 (ω3ω6), C22:4

The significant metabolites are divided into their clusters indicative of their trend and all had an ANOVA *p* value <0.05

### Metabolite clusters showing a “stable” increased or decreased trend over the four clinical phases of chronic HBV

Clusters 6 (Fig. 3a) and 8 (Fig. 3b) represent metabolites significantly altered in the IT phase compared with HCs and whose alteration persisted throughout the progression of chronic HBV infection with stable increased or decreased trends. Cluster 6 contained seven plasmalogen phospholipid and lysophospholipid species, of which three were significantly up-regulated in the IT phase compared with the HCs, having sustained increased levels across the four clinical phases. We named this cluster the “ROS-plasmalogen cluster”. Four non-enzymatic route oxylipins (10-, 11-, 13-, and 14-HDoHEs) grouped within cluster 6 indicate maintained oxidative stress experienced during the progression of chronic HBV infection. Interestingly, the choline catabolic metabolites betaine and sarcosine were also in the ROS-plasmalogen cluster, which may suggest some degree of choline metabolism perturbation. The amine metabolites carnitine and ornithine were identified as significantly changing over the clinical phases (Table 4). Carnitine is the free fatty acid transporter metabolite and indicates significant dysregulation during chronic HBV infection (*p* < 0.001). The IT and IC phases had lower carnitine levels compared with the IA and ENEG phases, reflecting increased energy requirements during these phases. Ornithine is a urea cycle intermediate together with citrulline (cluster 9, *p* = 0.006), indicating a dysregulated urea cycle, worsening with the progression of chronic HBV infection as both had their highest levels in the ENEG phase.

Cluster 8 represents the opposite trend, showing a stable decline of metabolite levels across the four clinical phases compared with HCs. Cluster 8 was labeled the “choline phospholipid cluster” as it comprises 14 phosphatidylcholines and 21 lysophosphatidylcholines. The strong initial reduction of choline phospholipids, together with the subsequent maintenance of the reduced levels over the four clinical phases, hints towards an imprinted metabolic alteration. Only the *sn1* and *sn2* lysophosphatidylcholine C20:3 species showed significant fluctuations during the progression of the clinical phases (Table 4). Three amines, including saccharopine, glutathione and γ-glutamyl glutamine, had stable decreased levels compared with HCs. The glutathione levels reflect diminished antioxidant capacity during chronic HBV, supporting the increased levels of lipid peroxidation products observed within cluster 6.

### Metabolite clusters showing a trend for increasing over the clinical phases of chronic HBV

From the 24 clusters, clusters 10 (Fig. 3c) and 15 (Fig. 3d) were the most dense, containing 47 and 42 metabolites, respectively. Both clusters showed an increasing trend across the four clinical phases of chronic HBV infection, with their defining difference observed when taking the HCs into account.

Cluster 10 shows subtle changes between the HCs and the IT phase but significantly increased during the progression of chronic HBV infection to levels well above HC levels in the ENEG phase. Cluster 10 is dominated by 13 amino acids and 17 triglyceride species, composed of long and very long acyl species with a combined acyl chain length of between C54 and C60. Thus, this cluster is called the “very long chain triglyceride cluster”. During the progression over the four clinical phases, 9 of the 17 triglycerides were identified as significantly increasing, revealing a perturbed triglyceride metabolism (Table 4). The essential amino acids methionine and phenylalanine were identified as significantly changing over the clinical phases and have both been identified as markers of hepatic injury and had their highest levels in the ENEG phase [31–33]. Choline, identified as significant and in cluster 10, is an integral part of glycerophospholipid and sphingolipid metabolism, acting as a functional lipid moiety. The increasing choline levels oppose that of its elevated catabolic metabolites betaine and sarcosine (cluster 6).

Cluster 15 includes metabolites that were significantly reduced in the IT phase compared with the HCs, after which metabolite levels started to increase back to HC levels. More than half of the 42 metabolites in cluster 15 are triglycerides, with a combined acyl chain length ranging from C42 to C55, effectively labeling this the “long chain triglyceride cluster”. These results show a biphasic triglyceride response during chronic HBV infection. Three of the five clustered lysophosphatidylethanolamines species, including C18:1, C20:3, and C20:4, were subsequently identified as significantly increasing over the four clinical phases, with levels almost resembling the HCs in the ENEG phase. These results also show that lysophosphatidylethanolamines (cluster 15) respond differently during chronic HBV infection to lysophosphatidylcholines (cluster 8).

### Metabolite clusters correlating to ALT and HBV DNA levels

During chronic HBV infection, ALT levels (Fig. 1a) were highest during the IA and ENEG phases, whereas HBV DNA levels (Fig. 1b) were high in the IT, IA, and ENEG phases and lowest in the IC phase. Clusters 13 (Fig. 3e) and 23 (Fig. 3f) represented metabolites correlating to ALT and HBV DNA levels, respectively.

Kynurenine identified in cluster 13 correlated to ALT levels and had significantly higher levels in the IA and ENEG phases compared with the IT and IC phases (Table 4). Kynurenine and its precursor amino acid tryptophan (cluster 15) were both also identified as significantly down-regulated during the IT phase compared with the HCs. Due to these fluctuating levels during the progression of chronic HBV, we inspected the tryptophan/kynurenine ratio relating to indoleamine 2,3-dioxygenase (IDO) activity. The tryptophan/kynurenine ratio had a *p* value of 0.038 across the five groups with a reduced IDO ratio in the IT, IA, and IC phases of chronic HBV infection compared with an ENEG phase ratio comparable to that of the HCs, implying altered IDO regulation during the first three phases of chronic HBV infection.

Cluster 23, reflecting HBV DNA levels during chronic HBV infection, consisted solely of five acyl-carnitines and four free fatty acids. The long chain free fatty acids C20:3 (ω3/ω6) and C22:4, together with decanoylcarnitine, had significantly lower levels in the IC phase compared with the other clinical phases, possibly correlating to HBV-induced secreted phospholipases [34, 35].

## Discussion

The present study is the first targeted and biology driven metabolomics profiling of chronic HBV infection, characterizing the natural progression through its distinct clinical phases. With the liver being the central organ in nutritional regulation and metabolism, it is not surprising that chronic HBV infections have been shown to induce multiple metabolic alterations in lipid metabolism of the host [15–21]. However, these studies have not addressed and positioned metabolic changes in relation to the progression of chronic HBV disease.

**Fig. 4:**
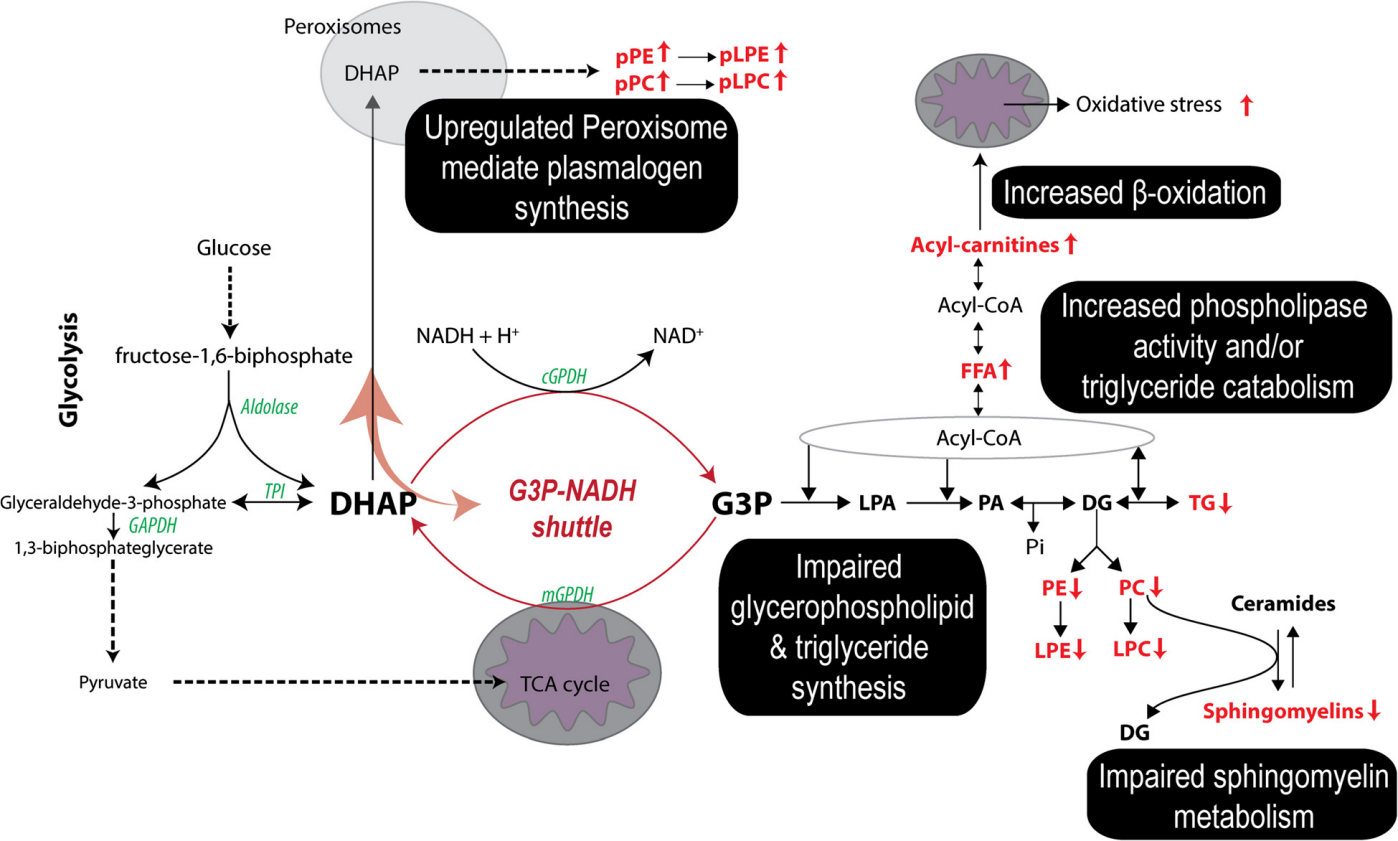
Metabolic alterations identified in the IT phase of chronic HBV. Increased levels of DHAP-derived plasmalogen phospholipid species, free fatty acids, and acyl carnitines were found. A significant decrease in glycerophospholipids, triglycerides, and sphingomyelins was found, suggesting the HBV hijacking of the cytosolic glycerol-3-phosphate dehydrogenase (*GPDH*) enzyme, favoring the synthesis of plasmalogen lipid species. Detected metabolic species are highlighted in *red* with the *arrow* indicating its trend. *DHAP* dihydroxyacetone phosphate, *G3P* glycerol-3-phosphate, *LPA* lysophosphatidic acid, *PA* phosphatidic acid, *DG* diacylglycerol, *PE* phosphatidylethanolamine, *PC* phosphatidylcholine, *LPE* lysophosphatidylethanolamine, *LPC* lysophosphatidylcholine, *TG* triglyceride, *FFA* free fatty acids, *pPE* plasmalogen phosphatidylethanolamine, *pPC* plasmalogen phosphatidylcholine, *pLPE* plasmalogen lysophosphatidylethanolamine, *pLPC* plasmalogen lysophosphatidylcholine, *GAPDH* glyceraldehyde-3-phosphate dehydrogenase, *TPI* triosephosphate isomerase, *cGPDH* cytosolic glycerol-3-phosphate dehydrogenase, *mGPDH* mitochondrial glycerol-3-phosphate dehydrogenase

The IT phase is clinically characterized by high levels of HBV replication and minimal hepatic injury. We now show that this phase exhibits major lipid alterations, with increased free fatty acids, acyl-carnitines, and plasmalogens concurrent with decreased triglyceride, phospholipid (ester-linked), and sphingomyelin levels (Fig. 4). The glycolysis intermediate dihydroxyacetone phosphate (DHAP) is the precursor metabolite via glycerol-3-phosphate (G3P) for the de novo glycerophospholipids and triglyceride synthesis pathways. Alternatively, DHAP can also be transported to the peroxisomes where it is the precursor for the synthesis of vinyl ether-linked plasmalogen phospholipids. The DHAP to G3P reaction is catalyzed by glycerol-3-phosphate dehydrogenase (GPDH) and is known as the G3P–NADH shuttle, simultaneously converting NADH to NAD^+^ necessary during the glycolysis cycle. Our results suggest that HBV hijacks this G3P–NADH shuttle, resulting in reduced levels of glycerophospholipids, lysoglycerophospholipids, triglycerides, and sphingomyelins. The secretion of glycerophospholipids from the liver is the main contributor to their serum levels, substantiating the hepatic metabolic fingerprint and the link to HBV activity [36]. Plasmalogen phosphatidylcholine has been identified as the preferred lipid species in the viral envelope and surface antigen particles of HBV, accounting for approximately 60 % of total lipid content [37]. Additionally, Li et al. [16] reported the upregulation of mRNA transcripts in the phosphatidylcholine biosynthesis pathway during HBV infection in HepG2 cell lines and its necessity for HBV replication. Collectively, the observed increase in choline plasmalogen levels reveals the metabolic engineering capacity of HBV and supports our hypothesis of HBV hijacking of the G3P–NADH shuttle. Importantly, this altered lipid profile of down-regulated phospholipids and increased plasmalogens persists during the progression of chronic HBV infection (Fig 3a, b), even during the IC phase where virtually no HBV replication is taking place, implying a HBV-induced metabolic imprinting effect. Although other reasons could be used to explain metabolic observations (such as an altered metabolic flux or consumption rate of metabolites and even diet), the use of pattern analyses and the progressive nature of our clinical phase-defined chronic HBV samples provide concrete evidence to illustrate an altered metabolic state.

During the natural progression of chronic HBV, we measured increased choline, methionine, and very long acyl chain triglyceride levels together with reduced phosphatidylcholine and lysophosphatidylcholine levels, indicating a perturbed choline metabolism. Dietary choline and methionine depravation is strongly linked to the development of steatosis, non-alcoholic fatty liver disease, cirrhosis, and HCC [38–41]. During conditions of choline restriction, the reduced levels of phosphatidylcholine, a critical component of the very low density lipoprotein particle, impair hepatic lipoprotein synthesis and result in the accumulation of free triglycerides within hepatocytes [42, 43]. Decreased phosphatidylcholine species in the presence of high choline levels during the IA, IC, and ENEG phases support the permanent G3P–NADH shuttle hijacking hypothesis, impairing lipoprotein synthesis during chronic HBV, while also explaining the accumulation of triglycerides. Even with decreased levels of long chain free fatty acids in the IC phase, no attenuation of long chain triglyceride levels was observed in this phase. The stable elevated levels of betaine, sarcosine, and methionine indicate enhanced choline catabolism, while increased levels of methionine are also reflective of hepatic injury [32, 33]. Previous studies demonstrated that host factors were responsible for the development of steatosis rather than viral factors [44, 45]. Our metabolomics data suggest that initiation of steatosis may be a consequence of HBV hijacking of the host’s glycerophopholipid metabolism, as liver fat content closely correlates with serum triglyceride levels [46, 47].

**Fig. 5:**
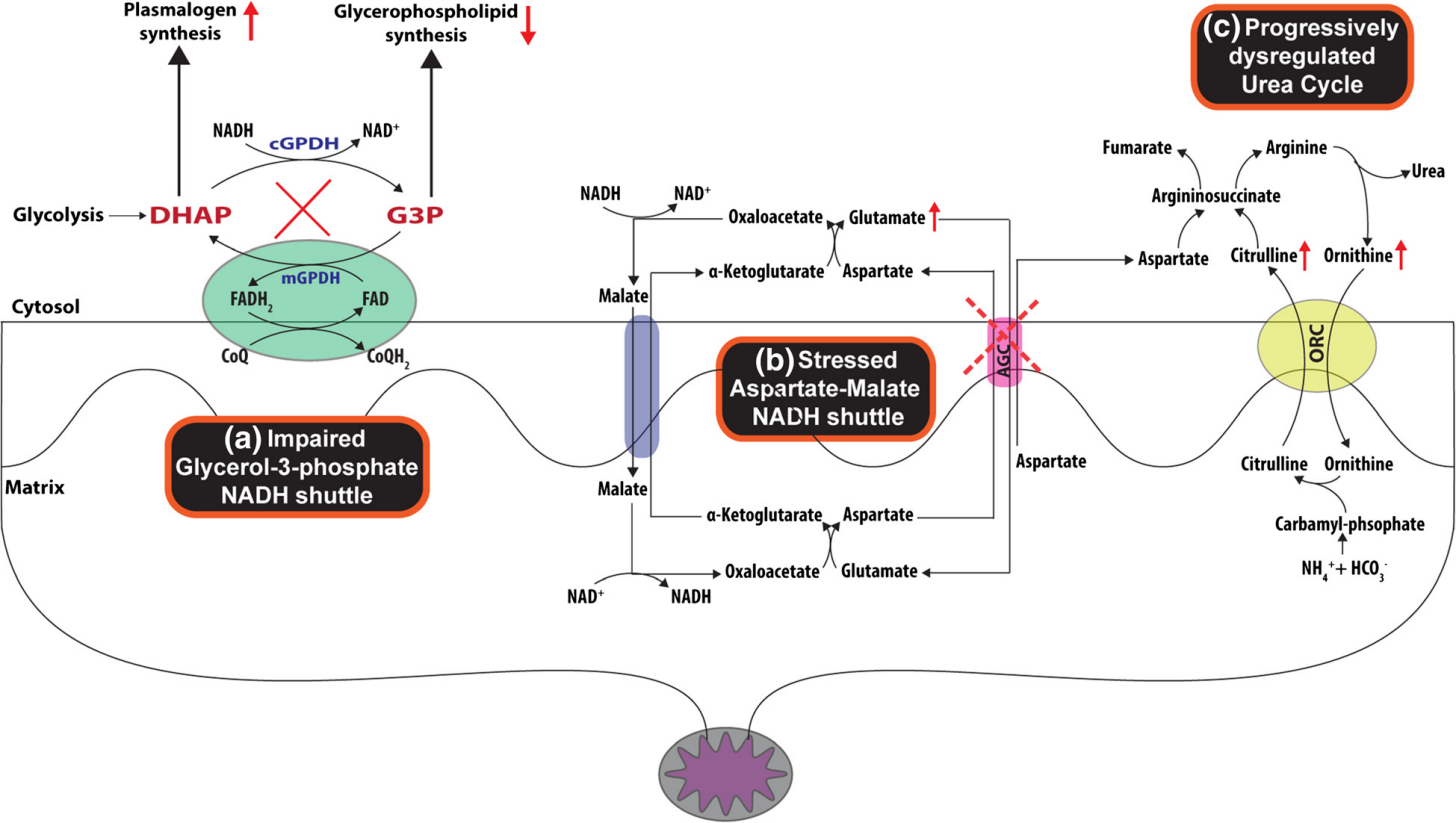
Cellular NADH shuttles and the urea cycle. The interplay between **a** the reduced G3P–NADH shuttle and **b** a stressed aspartate–malate NADH shuttle and its influence on **c** the urea cycle. The aspartate transporter AGC (aka citrin) facilitates the transport of aspartate across the mitochondrial membrane to the cytosol where it binds to citrulline in the urea cycle to help detoxify ammonium. Ineffective aspartate transport will lead to the accumulation of glutamate, citrulline, and ornithine identified in the IC and ENEG clinical phases of HBV infection

Another metabolic pathway with increased activity reflective of the natural progression of chronic HBV infection is composed of urea cycle intermediates: enhanced levels of citrulline and ornithine were detected in the IC and ENEG phases, respectively. The urea cycle is predominantly active in hepatocytes and responsible for detoxifying ammonia. Moreover, it has an intimate relationship with the aspartate–malate NADH shuttle functioning across the mitochondrial membrane. One could speculate that the HBV hijacking of the G3P–NADH shuttle, as explained above, will affect the redox status (NADH/NAD^+^) of the cell and cause an up-regulation/stress of the malate–aspartate NADH/NAD^+^ shuttle to rectify this imbalance (Fig. 5). The mitochondrial transporter citrin (AGC), encoded by the gene SLC25A13, is responsible for the transport of aspartate and glutamate during aspartate–malate NADH shuttling. Cytosolic aspartate binds to citrulline to form the urea cycle intermediate argininosuccinate, which can be converted to arginine, which in turn is converted to ornithine with the release of urea. We detected increased levels of citrulline, ornithine and glutamate, all implicating impaired aspartate transport, and may reflect reduced integrity and functioning of the mitochondrial citrin transporter. A dysregulated urea cycle precedes the histological manifestations of irreversible liver damage [48], and thus might be a prediction marker for chronic HBV progression and severity.

Collectively, the data presented here comprise the first metabolic study on the natural progression of chronic HBV infection using patient samples. We found impaired choline glycerophospholipid metabolism across the four chronic HBV clinical phases, together with increasing triglyceride and urea cycle intermediate levels as a liver metabolic fingerprint of the progression of chronic HBV infection. This metabolic fingerprint relates to HBV’s hijacking of the G3P–NADH shuttle, a key player in the plasmalogen, choline, and glycerophospholipid metabolic pathways, and its potential as a therapeutic target deserves further investigation. Elegant work done by Zeissig et al. [34] demonstrated the role of lysophospholipids as endogenous antigenic lipid species able to illicit protective immunological responses, which enhanced HBV clearance during acute infection. This may imply that, in addition to redirecting host lipid metabolism to produce the plasmalogens, hijacking of glycerophospholipid metabolism during acute HBV infection could act as a switch to determine HBV clearance or persistence. Furthermore, the diabetes drug metformin, which inhibits mitochondrial GPDH [49], part of the G3P–NADH shuttle, was found to inhibit HBV protein production and replication [50]. These findings substantiate the therapeutic value of the G3P–NADH shuttle in chronic HBV infection.

## Conclusions

The present study provides many insights and leads to design follow-up studies and, at the same time, highlights the need for a systems biology approach to better understand chronic HBV infection. We identified liver-related metabolic and injury perturbations, which reflect the natural progression of the disease. The altered glycerophospholipid metabolism in the IT phase attributed to the HBV hijacking of the G3P–NADH shuttle has an intimate relationship with the persistent lipid dysregulation observed in the IA, IC, and ENEG clinical phases. Increased levels of the very long chain triglycerides in the IA phase and urea cycle intermediates in the IC phase highlight the risk for developing secondary liver complications during chronic HBV infection. These metabolites might prove useful as markers of disease progression and severity.

## Abbreviations

ALT, alanine aminotransferase; DHAP, dihydroxyacetone phosphate; ENEG, HBeAg-negative; G3P, glycerol-3-phosphate; GPDH, glycerol-3-phosphate dehydrogenase; HBeAg, HBV envelope antigen; HBsAg, HBV surface antigen; HBV, hepatitis B virus; HC, healthy control; HCC, hepatocellular carcinoma; IA, immune active; IC, inactive carrier; IDO, indoleamine 2,3-dioxygenase; IT, immune tolerant; LC–MS, liquid chromatography–mass spectrometry; QC, quality control.

## Additional files

Additional file 1: Supplementary methods. (DOCX 33 kb) Additional file 2: Supplementary Figures S1 and S2 and Tables S1 and S3. (DOCX 397 kb) Additional file 3: Supplementary Table S2. (XLSX 38 kb)
