# Supplementary material for: Metabolic characterization of the natural progression of chronic hepatitis B
Source: Genome Med. 2016 Jun 10;8:64. doi: 10.1186/s13073-016-0318-8 (PMC4902991; doi:10.1186/s13073-016-0318-8)
Supplement: Additional file 1: — Supplementary methods. (DOCX 33 kb) [file 13073_2016_318_MOESM1_ESM.docx]

**Additional file 1: Supplemental methods**

**Metabolic characterisation of the natural progression of chronic hepatitis B.**

Johannes C. Schoeman^1,2^, Jun Hou^3^, Amy C. Harms^1,2^, Rob J. Vreeken^1,2,#^, Ruud Berger^1,2^, Thomas Hankemeier^1,2,*^, and Andre Boonstra^3,*,ᶴ^

^1^ Department of Analytical Biosciences, Leiden Academic Center for Drug Research, Leiden University, Einsteinweg 55, 2333 CC, Leiden, The Netherlands.

^2^ Netherlands Metabolomics Centre, Leiden University, Einsteinweg 55, 2333 CC, Leiden, The Netherlands.

^3^ Department of Gastroenterology and Hepatology, Erasmus University Medical Center, Rotterdam, Wytemaweg 80, 3015 CE, The Netherlands.

# * Both authors contributed equally

Current Address

^#^Discovery Sciences, Janssen R&D, Turnhoutseweg 30, 2340 Beerse, Belgium

# ᶴCorresponding author:

Andre Boonstra,
Department of Gastroenterology and Hepatology
Erasmus MC – University Medical Center Rotterdam
Wytemaweg 80, Room Na-1011
3015 CE Rotterdam, the Netherlands
Phone: +31 10 7035944, Fax: +31 10 7032793
E-mail: [p.a.boonstra@erasmusmc.nl](mailto:p.a.boonstra@erasmusmc.nl)

1. **Biogenic amine profiling**

The amine platform covers amino acids and biogenic amines employing an Accq-tag derivatization strategy adapted from the protocol supplied by Waters. 5 μL of each serum sample was spiked with an internal standard solution, thiol amines are released from proteins and converted to reduced using TCEP. Then proteins are precipitated by the addition of methanol. The supernatant was transferred to a new eppendorf tube and taken to dryness in a speedvac. The residue was reconstituted in borate buffer (pH 8.5) with AQC reagent. After reaction, the vials were transferred to an autosampler tray and cooled to 10°C until the injection. 1.0 μL of the reaction mixture was injected into the UPLC-MS/MS system.

**1.1 Equipment**

An ACQUITY UPLC system with autosampler (Waters, Etten-Leur, The Netherlands) was coupled online with a Xevo Tandem quadrupole mass spectrometer (Waters) operated using QuanLynx data acquisition software (version 4.1; Waters). The samples were analyzed by UPLC-MS/MS using an Accq-Tag Ultra column (Waters). The Xevo TQ was used in the positive-ion electrospray mode and all analytes were monitored in Multiple Reaction Monitoring (MRM) using nominal mass resolution.

**1.2 List of Detected Metabolites**

| **Metabolite** | **Chemical formula** | **HMDB ID** | **InChI Key** |
| --- | --- | --- | --- |
| 3-Aminoisobutyric acid | C4H9NO2 | HMDB00452 | QWCKQJZIFLGMSD-VKHMYHEASA-N |
| 4-Hydroxyproline | C5H9NO3 | HMDB00725 | PMMYEEVYMWASQN-DMTCNVIQSA-N |
| Alanine | C3H7NO2 | HMDB00161 | QNAYBMKLOCPYGJ-REOHCLBHSA-N |
| Alpha-aminobutyric acid | C4H9NO2 | HMDB03911 | QCHPKSFMDHP*SN*R-UHFFFAOYSA-N |
| Arginine | C6H14N4O2 | HMDB00517 | ODKSFYDXXFIFQN-BYPYZUCNSA-N |
| Asparagine | C4H8N2O3 | HMDB00168 | DCXYFEDJOCDNAF-REOHCLBHSA-N |
| Aspartic acid | C4H7NO4 | HMDB00191 | CKLJMWTZIZZHCS-REOHCLBHSA-N |
| Beta-alanine | C3H7NO2 | HMDB00056 | UCMIRNVEIXFBKS-UHFFFAOYSA-N |
| Citrulline | C6H13N3O3 | HMDB00904 | RHGKLRLOHDJJDR-BYPYZUCNSA-N |
| Cystathionine | C7H14N2O4S | HMDB00099 | ILRYLPWNYFXEMH-WHFBIAKZSA-N |
| Cysteine | C3H7NO2S | HMDB00574 | XUJNEKJLAYXESH-REOHCLBHSA-N |
| Ethanolamine | C2H7NO | HMDB00149 | HZAXFHJVJLSVMW-UHFFFAOYSA-N |
| gamma-Glutamylglutamine | C10H17N3O6 | HMDB11738 | JBFYFLXEJFQWMU-WDSKDSINSA-N |
| Glutamic acid | C5H9NO4 | HMDB00148 | WHUUTDBJXJRKMK-VKHMYHEASA-N |
| Glutamine | C5H10N2O3 | HMDB00641 | ZDXPYRJPNDTMRX-VKHMYHEASA-N |
| Glutathione | C10H17N3O6S | HMDB00125 | RWSXRVCMGQZWBV-WDSKDSINSA-N |
| Glycine | C2H5NO2 | HMDB00123 | DHMQDGOQFOQNFH-UHFFFAOYSA-N |
| Glycylglycine | C4H8N2O3 | HMDB11733 | YMAWOPBAYDPSLA-UHFFFAOYSA-N |
| Histidine | C5H9N3 | HMDB00870 | NTYJJOPFIAHURM-UHFFFAOYSA-N |
| Isoleucine | C6H13NO2 | HMDB00172 | AGPKZVBTJJNPAG-WHFBIAKZSA-N |
| Kynurenine | C10H12N2O3 | HMDB00684 | YGPSJZOEDVAXAB-QMMMGPOBSA-N |
| Leucine | C6H13NO2 | HMDB00687 | ROHFNLRQFUQHCH-YFKPBYRVSA-N |
| Lysine | C6H14N2O2 | HMDB00182 | KDXKERNSBIXSRK-YFKPBYRVSA-N |
| Methionine | C5H11NO2S | HMDB00696 | FFEARJCKVFRZRR-BYPYZUCNSA-N |
| Methionine sulfoxide | C5H11NO3S | HMDB02005 | QEFRNWWLZKMPFJ-UHFFFAOYSA-N |
| O-Phosphoethanolamine | C2H8NO4P | HMDB00224 | SUHOOTKUPISOBE-UHFFFAOYSA-N |
| Ornithine | C5H12N2O2 | HMDB00214 | AHLPHDHHMVZTML-BYPYZUCNSA-N |
| Phenylalanine | C9H11NO2 | HMDB00159 | COLNVLDHVKWLRT-QMMMGPOBSA-N |
| Pipecolic acid | C_6_H_11_NO_2_ | HMDB00716 | HXEACLLIILLPRG-YFKPBYRVSA-N |
| Saccharopine | C11H20N2O6 | HMDB00279 | ZDGJAHTZVHVLOT-YUMQZZPRSA-N |
| Sarcosine | C_3_H_7_NO_2_ | HMDB00271 | FSYKKLYZXJ*SN*PZ-UHFFFAOYSA-N |
| Serine | C3H7NO3 | HMDB00187 | MTCFGRXMJLQNBG-REOHCLBHSA-N |
| s-Methylcysteine | C4H9NO2S | HMDB02108 | IDIDJDIHTAOVLG-VKHMYHEASA-N |
| Taurine | C2H7NO3S | HMDB00251 | XOAAWQZATWQOTB-UHFFFAOYSA-N |
| Threonine | C4H9NO3 | HMDB00167 | AYFVYJQAPQTCCC-GBXIJSLDSA-N |
| Tryptophan | C11H12N2O2 | HMDB00929 | QIVBCDIJIAJPQS-VIFPVBQESA-N |
| Tyrosine | C9H11NO3 | HMDB00158 | OUYCCCASQSFEME-QMMMGPOBSA-N |
| Valine | C5H11NO2 | HMDB00883 | KZ*SN*JWFQEVHDMF-BYPYZUCNSA-N |

^* HMDB Human metabolome database^

1. **Positive lipid profiling**

Positive lipids were extracted with isopropyl alcohol (IPA). In short, 1000 μL IPA containing calibrant and internal standards both at C4 levels were added to 10 μL serum to precipitate proteins. After centrifugation (12.100 rpm, 10 mins, at RT) supernatant containing the lipids is transferred to vials for LC-MS analysis. In total 2.5 μL was injected for analysis.

**2.1 Equipment**

Chromatographic separation was achieved on an ACQUITY UPLC™ HSS T3 column (1.8 μm, 2.1 x 100mm) with a flow of 0.4 mL/min over a 16 min gradient. The lipid analysis is performed on a UPLC-ESI-Q-TOF (Agilent 6530, Jose, CA, USA) high resolution mass spectrometer using reference mass correction. Lipids were detected in full scan in the positive ion mode.

- 1. **List of** **Detected Metabolites**

| **Lipid Class** | **Lipidmaps** | **Metabolite species** | **Amount (n)** |
| --- | --- | --- | --- |
| Cholesteryl ester (CE) | ST0102 | C18:1; C18:2; C18:3; C20:4; C20:5; C22:6 | 6 |
| Ceremides (Cer) | SP02 | d18:1/24:0; d18:1/24:1 | 2 |
| Diacylglycerol (DG) | GL0201 | C36:2; C36:3; C36:4 | 3 |
| Lysophosphatidylcholine (LPC) | GP0105 | C14:0; C16:0; C16:1; C18:0; C18:1; C18:2; C19:0; C20:3; C20:4; C20:5; C22:6 | 11 |
| Lysophosphatidylethanolamine (LPE) | GP0205 | C18:0 | 1 |
| Phosphatidylcholine (PC) | GP0101 | C32:0; C32:1; C32:2; C34:1; C34:2; C34:3; C34:4; C36:1; C36:2; C36:3; C36:4; C36:5; C36:6; C38:2; C38:3; C38:4; C38:5; C38:6; C38:7; C40:4; C40:5; C40:6; C40:7; C40:8 | 24 |
| Phosphatidylethanolamine (PE) | GP0201 | C34:2; C36:3; C36:4; C38:2; C38:4; C38:6 | 6 |
| Plasmalogen Lysophosphatidylcholine (pLPC) | GP0106 | C16:1; C18:1 | 2 |
| Plasmalogen Phosphatidylcholine (pPC) | GP0102 | C34:1; C34:2; C34:3; C36:2; C36:3; C36:4; C36:5; C36:6; C38:4; C38:5; C38:6; C40:6; C42:6; C44:5 | 14 |
| Plasmalogen Phosphatidylethanolamine (pPE) | GP0202 | C36:5; C38:5; C38:7 | 3 |
| Sphingomyelins (SM) | SP0301 | d18:1/14:0; d18:1/15:0; d18:1/16:0; d18:1/16:1; d18:1/18:0; d18:1/18:1; d18:1/18:2; d18:1/20:0; d18:1/20:1; d18:1/21:0; d18:1/22:0; d18:1/22:1; d18:1/23:0; d18:1/23:1; d18:1/24:0; d18:1/24:1; d18:1/24:2; d18:1/25:0; d18:1/25:1 | 19 |
| Triglycerides (TG) | GL0301 | C42:1; C44:2; C46:1; C46:2; C46:3; C48:1; C48:2; C48:3; C48:4; C50:0; C50:1; C50:2; C50:3; C50:4; C50:5; C51:1; C51:2; C51:3; C51:4; C52:1; C52:2; C52:3; C52:4; C52:5; C52:6; C54:1; C54:2; C54:3; C54:4; C54:5; C54:6; C54:7; C55:2; C55:3; C56:2; C56:3; C56:4; C56:5; C56:6; C56:7; C56:8; C58:3; C58:4; C58:5; C58:8; C58:10; C60:2; C60:3; | 48 |

1. **Polar negative lipid profiling**

Polar lipid extraction is performed by methanol extraction. Briefly, 440 μL methanol containing internal standard is added to 20 μL serum. After centrifugation, the protein is precipitated and the lipid containing supernatant is transferred to clean Eppendorf tubes and the solvent is evaporated using a Speedvac. The dried lipids were reconstituted in 145 μL isopropanol, 0.1% formic acid and transferred to autosampler vials. In total 8 μL was injected for analysis.

**3.1 Equipment**

Chromatographic separation was achieved on an ACQUITY UPLC™ HSS T3 column with a flow of 0.4 ml/min over 15 min gradient. Free fatty acids and lyso-phospholipid content is analyzed with a UPLC-ESI-Q-TOF (Agilent 6530, Jose, CA, USA) high resolution mass spectrometer using reference mass correction. Polar lipids were detected in full scan in the negative ion mode.

**3.2 List of Detected Metabolites**

| **Lipid Class** | **Lipidmaps** | **Metabolite species** | **Amount (n)** |
| --- | --- | --- | --- |
| Free fatty acids (FA) | FA01 | C14:0; C15:0; C16:1; C17:1; C18:1; C18:2; C18:3-(w3w6); C20:1; C20:2; C20:3-(w9); C20:3-(w3w6); C20:4-(w6); C20:5-(w3); C22:4; C22:5-(w3); C22:5-(w6); C22:6; C24:1 | 18 |
| Lysophosphatidylcholine (LPC) | GP0105 | *sn1:* C14:0; C15:0; C16:0; C16:1; C18:0; C18:1; C18:2; C18:3-(w3w6); C18:3-(w3w6); C19:0; C20:1; C20:3-(w9); C20:3-(w3w6); C20:4; C20:5; C22:4; C22:5-(w3); C22:5-(w6); C22:6  *sn2:* C14:0; C16:0; C16:1; C18:0; C18:1; C18:2; C18:3-(w3w6); C18:3-(w3w6); C20:3-(w3w6); C20:4; C20:5; C22:6 | 31 |
| Lysophosphatidylethanolamine (LPE) | GP0205 | C16:0; C18:0; C18:1; C18:2; C20:3-(w3w6); C20:4; 22:5-(w3); C22:6 | 8 |
| Plasmalogen Lysophosphatidylcholine (pLPC) | GP0106 | C16:0; C18:0; C18:1; C18:2 | 4 |

1. **Acyl-carnitine profiling**

The acyl-carnitine platform covers acyl-carnitines as well as Trimethylamine-N-oxide, Choline, Betaine, Deoxycarnitine and Carnitine. 10 μL of each serum sample was spiked with an internal standard solution, followed by deproteination by addition of methanol. The supernatant was transferred to an autosampler vial. The vials were transferred to an autosampler tray and cooled to 10°C until the injection. 1.0 μL of the reaction mixture was injected into the UPLC-MS/MS system.

**4.1 Equipment**

An ACQUITY UPLC system with autosampler (Waters, Etten-Leur, The Netherlands) was coupled online with a Xevo Triple quadrupole mass spectrometer (Waters) operated using Masslynx data acquisition software (version 4.1; Waters). The samples were analyzed by UPLC-MS/MS using an Accq-Tag Ultra column (Waters). The Xevo TQ was used in the positive-ion electrospray mode and all analytes were monitored in Multiple Reaction Monitoring (MRM) using nominal mass resolution.

**4.2 List of Detected Metabolites**

| **Metabolite** | **Acyl length** | **HMDB* ID** | **InChIKey** |
| --- | --- | --- | --- |
| 2-Methylbutyroylcarnitine | C5:0 | HMDB41993 | YICAQFPUDACYGQ-UHFFFAOYSA-N |
| Betaine | C0 | HMDB00043 | KWIUHFFTVRNATP-UHFFFAOYSA-N |
| Butyrylcarnitine | C4:0 | HMDB02013 | LRCNOZRCYBNMEP-SECBINFHSA-N |
| Carnitine | C0 | HMDB00062 | PHIQHXFUZVPYII-ZCFIWIBFSA-N |
| Choline | C0 | HMDB00097 | OEYIOHPD*SN*JKLS-UHFFFAOYSA-N |
| Decanoylcarnitine | C10:0 | HMDB00651 | KETNUEKCBCWXCU-UHFFFAOYSA-N |
| Decenoylcarnitine | C10:1 | HMDB13205 | GOOOCIIXFLVRAG-UHFFFAOYSA-N |
| Deoxycarnitine | C0 | HMDB01161 | GNRKTORAJTTYIW-UHFFFAOYSA-N |
| Dodecenoylcarnitine | C12:1 | HMDB13326 | JEOZLTJHDSKQIT-CLVCIHKQSA-N |
| Hexadecenoylcarnitine | C16:1 | HMDB13207 | ZVOMLLYYTWFWOA-XPTLAUCJSA-N |
| Hexanoylcarnitine | C6:1 | HMDB13161 | JUAQYRSUSCWTQK-BQYQJAHWSA-N |
| Isobutyrylcarnitine | C4:0 | HMDB02013 | LRCNOZRCYBNMEP-SECBINFHSA-N |
| Isovalerylcarnitine | C5:0 | HMDB00688 | IGQBPDJNUXPEMT-UHFFFAOYSA-N |
| Lauroylcarnitine | C12:0 | HMDB02250 | FUJLYHJROOYKRA-QGZVFWFLSA-N |
| Linoleylcarnitine | C18:2 | HMDB06469 | MJLXQSQYKZWZCB-UTJQPWESSA-N |
| Myristoylcarnitine | C14:0 | HMDB05066 | PSHXNVGSVNEJBD-LJQANCHMSA-N |
| Nonaylcarnitine | C9:0 | HMDB13288 | MPSPNFAQQQMFLK-UHFFFAOYSA-N |
| Octanoylcarnitine | C8:0 | HMDB00791 | CXTATJFJDMJMIY-CYBMUJFWSA-N |
| Octenoylcarnitine | C8:1 | HMDB13324 | LOSHAHDSFZXVCT-MDZDMXLPSA-N |
| Oleylcarnitine | C18:1 | HMDB05065 | IPOLTUVFXFHAHI-SEYXRHQNSA-N |
| Palmitoylcarnitine | C16:0 | HMDB00222 | XOMRRQXKHMYMOC-OAQYLSRUSA-N |
| Propionylcarnitine | C3:0 | HMDB00824 | UFAHZIUFPNSHSL-UHFFFAOYSA-N |
| Stearoylcarnitine | C18:0 | HMDB00848 | YSVYWVPJJXVIFM-UHFFFAOYSA-N |
| Tetradecadienylcarnitine | C14:2 | HMDB13331 | REUVFPFCXKWJQA-UTJQPWESSA-N |
| Tetradecenoylcarnitine | C14:1 | HMDB02014 | NNCBVXBBLABOCB-SEYXRHQNSA-N |
| Trimethylamine-N-oxide | C0 | HMDB00925 | UYPYRKYUKCHHIB-UHFFFAOYSA-N |

* Human metabolome database

1. **Oxylipin profiling**

The oxylipin platform covers classical and non-classical eicosanoids from different poly unsaturated fatty acids (PUFA), including n-6 and n-3 PUFAs such as linoleic acid and arachidonic acid (both n-6) and eicosapentaenoic acid (EPA) and docosahexaenoic acid (DHA) (both n-3). 250 μL of each plasma sample were spiked with antioxidants and internal standard mix (Table 8), diluted and add to the SPE cartridge using a hydrophilic-lipophilic balance (HLB) (Oasis, Waters). Oxylipins were eluted with methanol and ethyl acetate. To concentrate, the eluate was gently dried under nitrogen stream and reconstituted in 50 μL of injection solvent (acetonitrile and methanol,1:1 v/v). In total 5 μL was injected for analysis.

**5.1 Equipment**

The HPLC was coupled to electrospray ionization on a triple quadrupole mass spectrometer (Agilent 6460, San Jose, CA, USA). Separation was done by HPLC (Agilent 1260, San Jose, CA, USA) using an Ascentis® Express column (2.1x150 mm, 2.7 μm particles; Supelco, Bellefonte, PA, USA) with 0.35 mL flow during 28 min gradient. Oxylipins were detected in negative ion mode using dynamic Multiple Reaction Monitoring (MRM).

**5.2 List of Detected Metabolites**

| **Metabolite** | **Systematic name** | **Formula** | **Lipid Maps ID** |
| --- | --- | --- | --- |
| 10-HDoHE | (+/-)-10-hydroxy-4Z,7Z,11E,13Z,16Z,19Z-docosahexaenoic acid | C22H32O3 | LMFA04000027 |
| 11-HDoHE | (+/-)-11-hydroxy-4Z,7Z,9E,13Z,16Z,19Z-docosahexaenoic acid | C22H32O3 | LMFA04000028 |
| 11-HETE | 11R-hydroxy-5Z,8Z,12E,14Z-eicosatetraenoic acid | C20H32O3 | LMFA03060028 |
| 12,13-DiHODE | (+/-)-12,13-dihydroxy-9Z,15Z-octadecadienoic acid | C18H32O4 | LMFA02000046 |
| 12,13-DiHOME | 12,13-dihydroxy-9Z-octadecenoic acid | C18H34O4 | LMFA01050351 |
| 12,13-EpOME | (+/-)-12(13)-epoxy-9Z-octadecenoic acid | C18H32O3 | LMFA02000038 |
| 12-HETE | 12-hydroxy-5Z,8Z,10E,14Z-eicosatetraenoic acid | C20H32O3 | LMFA03060088 |
| 12S-HEPE | 12S-hydroxy-5Z,8Z,10E,14Z,17Z-eicosapentaenoic acid | C20H30O3 | LMFA03070008 |
| 12S-HHTrE | 12S-hydroxy-5Z,8E,10E-heptadecatrienoic acid | C17H28O3 | LMFA03050002 |
| 13-HDoHE | (+/-)-13-hydroxy-4Z,7Z,10Z,14E,16Z,19Z-docosahexaenoic acid | C22H32O3 | LMFA04000029 |
| 13-HODE | 13S-hydroxy-9Z,11E-octadecadienoic acid | C18H32O3 | LMFA01050349 |
| 13-KODE | 13-keto-9Z,11E-octadecadienoic acid | C18H30O3 | LMFA02000016 |
| 14,15-DiHETrE | 14,15-dihydroxy-5Z,8Z,11Z-eicosatrienoic acid | C20H34O4 | LMFA03050010 |
| 14-HDoHE | (+/-)-14-hydroxy-4Z,7Z,10Z,12E,16Z,19Z-docosahexaenoic acid | C22H32O3 | LMFA04000030 |
| 15-HETE | 15S-hydroxy-5Z,8Z,11Z,13E-eicosatetraenoic acid | C20H32O3 | LMFA03060001 |
| 15S-HEPE | 15S-hydroxy-5Z,8Z,11Z,13E,17Z-eicosapentaenoic acid | C20H30O3 | LMFA03070009 |
| 15S-HETrE | 15S-hydroxy-8Z,11Z,13E-eicosatrienoic acid | C20H34O3 | LMFA03050007 |
| 17,18-DiHETE | (+/-)-17,18-dihydroxy-5Z,8Z,11Z,14Z-eicosatetraenoic acid | C20H32O4 | LMFA03060078 |
| 19,20-DiHDPA | (±)19,20-dihydroxy-4Z,7Z,10Z,13Z,16Z-docosapentaenoic acid | C22H34O4 | LMFA04000043 |
| 19,20-EpDPE | (+/-)-19(20)-epoxy-4Z,7Z,10Z,13Z,16Z-docosapentaenoic acid | C22H32O3 | LMFA04000038 |
| 20-HDoHE | (+/-)-20-hydroxy-4Z,7Z,10Z,13Z,16Z,18E-docosahexaenoic acid | C22H32O3 | LMFA04000033 |
| 5,6-DiHETrE | 5S,6S-dihydroxy-7E,9E,11Z,14Z-eicosatetraenoic acid | C20H32O4 | LMFA03060018 |
| 5-HETE | 5S-hydroxy-6E,8Z,11Z,14Z-eicosatetraenoic acid | C20H32O3 | LMFA03060002 |
| 5-HETrE | 5S-hydroxy-6E,8Z,11Z-eicosatrienoic acid | C20H34O3 | LMFA03050005 |
| 5S-HEPE | 5S-hydroxy-6E,8Z,11Z,14Z,17Z-eicosapentaenoic acid | C20H30O3 | LMFA03070010 |
| 8,9-DiHETrE | 8,9-dihydroxy-5Z,11Z,14Z-eicosatrienoic acid | C20H34O4 | LMFA03050006 |
| 9,10-DiHOME | 9,10-dihydroxy-12Z-octadecenoic acid | C18H34O4 | LMFA01050350 |
| 9,10-EpOME | (+/-)-9(10)-epoxy-12Z-octadecenoic acid | C18H32O3 | LMFA02000037 |
| 9,12,13-TriHOME | 9S,12S,13S-trihydroxy-10E-octadecenoic acid | C18H34O5 | LMFA02000014 |
| 9-HODE | 9S-hydroxy-10E,12Z-octadecadienoic acid | C18H32O3 | LMFA01050278 |
| 9-HOTrE | 9S-hydroxy-10E,12Z,15Z-octadecatrienoic acid | C18H30O3 | LMFA02000024 |
| LTB4 | 5S,12R-dihydroxy-6Z,8E,10E,14Z-eicosatetraenoic acid | C20H32O4 | LMFA03020001 |
| PGE2 | 9-oxo-11R,15S-dihydroxy-5Z,13E-prostadienoic acid; Prostin E2 | C20H32O5 | LMFA03010003 |
| PGF2a | 9S,11R,15S-trihydroxy-5Z,13E-prostadienoic acid | C20H34O5 | LMFA03010002 |
| TXB2 | 9S,11,15S-trihydroxy-thromboxa-5Z,13E-dien-1-oic acid | C20H34O6 | LMFA03030002 |
