# Supplementary material for: Metabolic characterization of the natural progression of chronic hepatitis B
Source: Genome Med. 2016 Jun 10;8:64. doi: 10.1186/s13073-016-0318-8 (PMC4902991; doi:10.1186/s13073-016-0318-8)
Supplement: Additional file 2: — Supplementary Figures S1 and S2 and Tables S1 and S3. (DOCX 397 kb) [file 13073_2016_318_MOESM2_ESM.docx]

**Additional file 2: Supporting information**

**Metabolic characterisation of the natural progression of chronic hepatitis B.**

Johannes C. Schoeman^1,2^, Jun Hou^3^, Amy C. Harms^1,2^, Rob J. Vreeken^1,2,#^, Ruud Berger^1,2^, Thomas Hankemeier^1,2,*^, and Andre Boonstra^3,*,ᶴ^

^1^ Department of Analytical Biosciences, Leiden Academic Center for Drug Research, Leiden University, Einsteinweg 55, 2333 CC, Leiden, The Netherlands.

^2^ Netherlands Metabolomics Centre, Leiden University, Einsteinweg 55, 2333 CC, Leiden, The Netherlands.

^3^ Department of Gastroenterology and Hepatology, Erasmus University Medical Center, Rotterdam, Wytemaweg 80, 3015 CE, The Netherlands.

# * Both authors contributed equally

Current Address

^#^Discovery Sciences, Janssen R&D, Turnhoutseweg 30, 2340 Beerse, Belgium

# ᶴCorresponding author:

Andre Boonstra,
Department of Gastroenterology and Hepatology
Erasmus MC – University Medical Center Rotterdam
Wytemaweg 80, Room Na-1011
3015 CE Rotterdam, the Netherlands
Phone: +31 10 7035944, Fax: +31 10 7032793
E-mail: [p.a.boonstra@erasmusmc.nl](mailto:p.a.boonstra@erasmusmc.nl)

**Figures**

| PCA scores plot |
| --- |
| 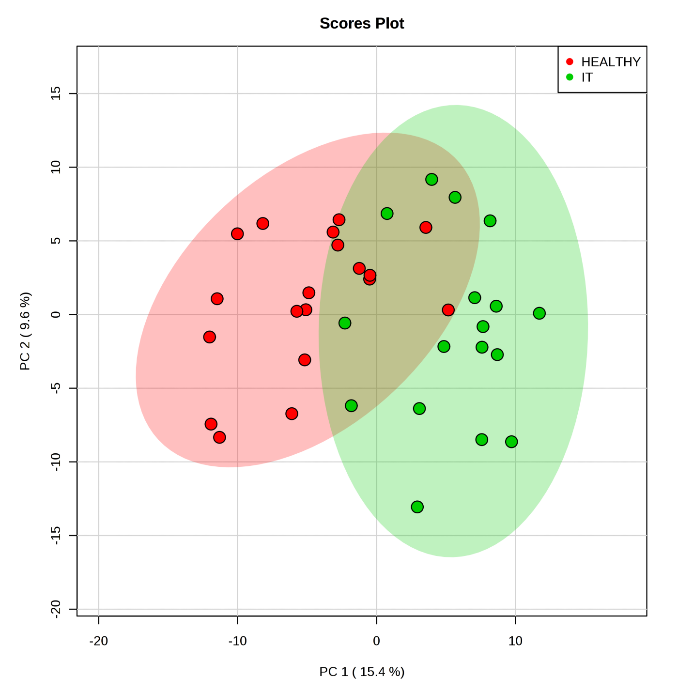  **Figure S1. HCs vs IT phase PCA analyses.** PCA analyses plotting PC1 vs. PC2 for the HCs (Red) versus the IT phase (Green), using the fused data set comprising of 37 cases and all 314 detected metabolites. Natural group clustering lead to partial differentiation between the two groups, indicating metabolic differences between them. |


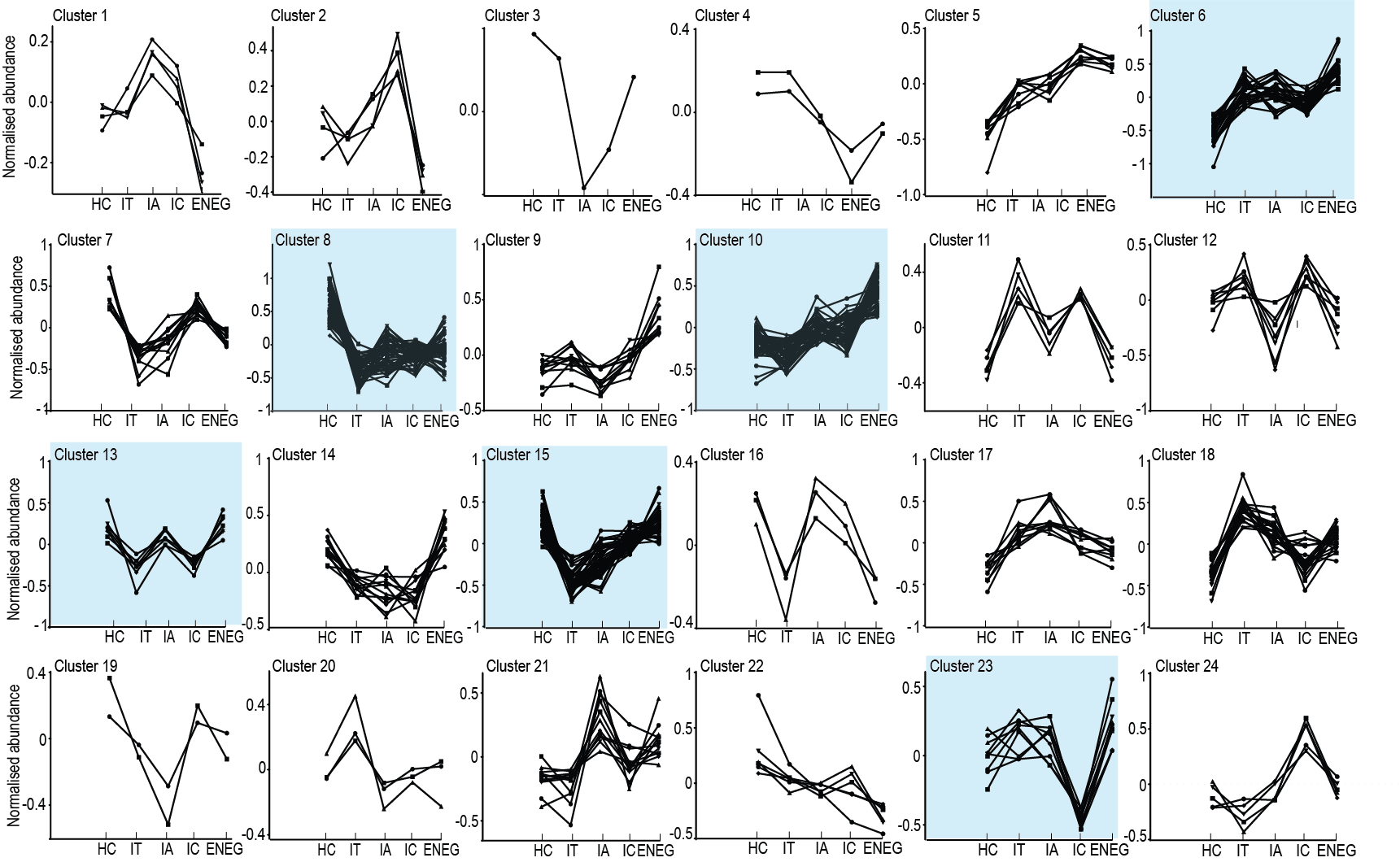


**Figure. S2. K-means clustering.** Metabolites are grouped into different classes by K-means clustering based on the changes of their abundance during the progression of chronic HBV. Expression pattern-based analysis was performed with all 314 metabolites and 88 patients. A K-means clustering was performed in MATLAB using *kmeans* on these metabolites in order to examine alterations in their measurement during the course of chronic HBV. Within each cluster metabolites present similar expression patterns, while different from metabolites in other clusters. The 24 clusters present the best partition of all 314 metabolites and are shown in the figure, with the blue highlighted clusters being discussed in the main text. The x-axis shows the healthy controls (HC) and the four clinical phases (IT, IA, IC, and ENEG). The y-axis is the abundance of metabolites normalized to the overall mean of the metabolite across all samples.

**Tables.**

**TABLE S1. Descriptive statistics of significant serum metabolites between HCs and the IT phase**. The table contains their corresponding statistical scores and descriptive statistics using normalised area ratios, ordered by metabolic class.

| Targeted Metabolomics Platform | | Metabolite | T-Test | False discovery rate | Fold change | Healthy Controls | IT Phase |
| --- | --- | --- | --- | --- | --- | --- | --- |
|  |  |  | *p*-value | *q*-value | (FC) | Mean ± SD | Mean ± SD |
|  | ***Phospholipids*** | | | | | | |
| Positive Lipids | | PC C32:1 | 0.0012 | 0.006 | 0.51 | 0.68 ± 0.44 | 0.35 ± 0.22 |
|  |  | PC C32:2 | < 0.0001 | < 0.001 | 0.52 | 0.19 ± 0.06 | 0.1 ± 0.04 |
|  |  | PC C34:1 | 0.0029 | 0.010 | 0.76 | 7.74 ± 1.9 | 5.91 ± 1.52 |
|  |  | PC C34:3 | 0.0001 | 0.001 | 0.64 | 0.67 ± 0.22 | 0.43 ± 0.14 |
|  |  | PC C34:4 | < 0.0001 | < 0.001 | 0.45 | 0.06 ± 0.02 | 0.03 ± 0.01 |
|  |  | PC C36:3 | < 0.0001 | 0.001 | 0.72 | 6.36 ± 1.65 | 4.62 ± 0.86 |
|  |  | PC C36:4 | 0.0006 | 0.003 | 0.75 | 7.2 ± 1.64 | 5.47 ± 1.1 |
|  |  | PC C36:6 | 0.0134 | 0.035 | 0.72 | 0.03 ± 0.01 | 0.02 ± 0.01 |
|  |  | PC C38:3 | 0.0063 | 0.018 | 0.7 | 1.37 ± 0.49 | 0.96 ± 0.37 |
|  |  | PE C38:4 | < 0.0001 | < 0.001 | 0.6 | 0.33 ± 0.1 | 0.2 ± 0.07 |
|  | ***Plasmalogens*** | | | | | | |
| Positive Lipids | | pPC C34:2 | 0.0003 | 0.002 | 1.37 | 0.29 ± 0.08 | 0.4 ± 0.1 |
|  |  | pPC C36:2 | 0.0005 | 0.003 | 1.25 | 0.09 ± 0.02 | 0.12 ± 0.02 |
|  |  | pPC C36:3 | 0.0005 | 0.003 | 1.3 | 0.07 ± 0.02 | 0.1 ± 0.02 |
|  |  | pPC C38:6 | 0.0025 | 0.009 | 1.6 | 0.16 ± 0.03 | 0.22 ± 0.08 |
|  |  | pPE C38:5 | 0.002 | 0.008 | 1.42 | 0.19 ± 0.06 | 0.28 ± 0.09 |
|  |  | pPE C38:7* | 0.0229 | 0.052 | 1.4 | 0.07 ± 0.02 | 0.1 ± 0.03 |
|  | ***Lysophospholipids*** | | | | | | |
| Negative Lipids | | LPE C18:0 | 0.0002 | 0.002 | 0.65 | 0.13 ± 0.03 | 0.08 ± 0.03 |
|  |  | LPE C18:1 | 0.0027 | 0.008 | 0.66 | 0.07 ± 0.03 | 0.05 ± 0.02 |
|  |  | LPE C18:2* | 0.025 | 0.052 | 0.71 | 0.14 ± 0.07 | 0.10 ± 0.05 |
|  |  | LPE C20:3 (w3w6) | 0.0002 | 0.001 | 0.54 | 0.008 ± 0.003 | 0.005 ± 0.002 |
|  |  | LPE C20:4 | 0.0008 | 0.003 | 0.72 | 0.079 ± 0.023 | 0.058 ± 0.015 |
|  |  | LPE C22:5 (w3)* | 0.0249 | 0.052 | 0.75 | 0.008 ± 0.003 | 0.006 ± 0.002 |
|  |  | *sn1* LPC C14:0 | < 0.0001 | < 0.001 | 0.47 | 0.19 ± 0.06 | 0.09 ± 0.03 |
|  |  | *sn1* LPC C15:0 | < 0.0001 | < 0.001 | 0.63 | 0.1 ± 0.02 | 0.06 ± 0.02 |
|  |  | *sn1* LPC C16:1 | 0.0002 | 0.001 | 0.71 | 0.35 ± 0.09 | 0.25 ± 0.07 |
|  |  | *sn1* LPC C18:1 | < 0.0001 | < 0.001 | 0.73 | 4.04 ± 0.67 | 2.99 ± 0.68 |
|  |  | *sn1* LPC C18:2 | 0.0081 | 0.02 | 0.79 | 8.0 ± 2.1 | 6.4 ± 1.6 |
|  |  | *sn1* LPC C18:3 (w3w6) | < 0.0001 | < 0.001 | 0.55 | 0.118 ± 0.041 | 0.065 ± 0.031 |
|  |  | *sn1* LPC C20:3 (w3w6) | < 0.0001 | < 0.001 | 0.53 | 0.82 ± 0.33 | 0.44 ± 0.15 |
|  |  | *sn1* LPC C20:3 (w9) | < 0.0001 | < 0.001 | 0.46 | 0.034 ± 0.017 | 0.015 ± 0.008 |
|  |  | *sn1* LPC C20:4 | 0.0003 | 0.001 | 0.69 | 1.84 ± 0.52 | 1.28 ± 0.35 |
|  |  | *sn1* LPC C22:4 | 0.008 | 0.02 | 0.67 | 0.053 ± 0.02 | 0.035 ± 0.01 |
|  |  | *sn1* LPC C22:5 (w6) | 0.0015 | 0.004 | 0.59 | 0.042 ± 0.019 | 0.025 ± 0.01 |
|  |  | *sn2* LPC C14:0 | < 0.0001 | < 0.001 | 0.46 | 0.04 ± 0.013 | 0.018 ± 0.007 |
|  |  | *sn2* LPC C16:1 | 0.0001 | < 0.001 | 0.69 | 0.068 ± 0.018 | 0.047 ± 0.013 |
|  |  | *sn2* LPC C18:1 | < 0.0001 | < 0.001 | 0.74 | 0.7 ± 0.11 | 0.52 ± 0.12 |
|  |  | *sn2* LPC C18:2 | 0.0058 | 0.015 | 0.77 | 1.59 ± 0.42 | 1.24 ± 0.32 |
|  |  | *sn2* LPC C18:3 (w3w6) | < 0.0001 | < 0.001 | 0.54 | 0.02 ± 0.01 | 0.01 ± 0.01 |
|  |  | *sn2 L*PC C20:3 (w3w6) | < 0.0001 | < 0.001 | 0.54 | 0.16 ± 0.06 | 0.09 ± 0.03 |
|  |  | *sn2* LPC C20:4 | 0.0049 | 0.013 | 0.73 | 0.35 ± 0.11 | 0.26 ± 0.07 |
|  | ***Lyso-Plasmalogens*** | | | | | | |
| Negative Lipids | | pLPC C16:0 | 0.0006 | 0.002 | 1.32 | 0.1 ± 0.03 | 0.13 ± 0.02 |
|  |  | pLPC C16:1 | 0.0035 | 0.011 | 1.21 | 0.03 ± 0.005 | 0.04 ± 0.006 |
|  |  | pLPC C18:1 | 0.0001 | 0.003 | 1.44 | 0.068 ± 0.018 | 0.088 ± 0.017 |
|  |  | pLPC C18:2 | 0.0017 | 0.005 | 1.32 | 0.014 ± 0.004 | 0.019 ± 0.004 |
|  | ***Triglycerides*** | | | | | | |
| Positive Lipids | | TG C42:1 | 0.0055 | 0.017 | 0.42 | 0.07 ± 0.05 | 0.03 ± 0.02 |
|  |  | TG C44:2 | 0.0016 | 0.007 | 0.49 | 0.11 ± 0.07 | 0.05 ± 0.02 |
|  |  | TG C46:1 | < 0.0001 | 0.001 | 0.44 | 0.73 ± 0.43 | 0.32 ± 0.11 |
|  |  | TG C46:2 | < 0.0001 | 0.001 | 0.4 | 0.37 ± 0.22 | 0.15 ± 0.06 |
|  |  | TG C46:3 | 0.0015 | 0.007 | 0.3 | 0.09 ± 0.06 | 0.03 ± 0.02 |
|  |  | TG C48:1 | 0.0002 | 0.002 | 0.4 | 1.93 ± 1.29 | 0.79 ± 0.57 |
|  |  | TG C48:2 | < 0.0001 | 0.001 | 0.4 | 1.44 ± 0.87 | 0.58 ± 0.33 |
|  |  | TG C48:3 | 0.0005 | 0.003 | 0.43 | 0.49 ± 0.3 | 0.21 ± 0.09 |
|  |  | TG C48:4* | 0.0233 | 0.052 | 0.52 | 0.1 ± 0.07 | 0.05 ± 0.03 |
|  |  | TG C50:0 | 0.0209 | 0.049 | 0.66 | 0.70 ± 0.43 | 0.46 ± 0.42 |
|  |  | TG C50:1 | 0.0017 | 0.007 | 0.51 | 4.05 ± 2.47 | 2.09 ± 1.67 |
|  |  | TG C50:2 | 0.0009 | 0.005 | 0.55 | 4.62 ± 2.27 | 2.55 ± 1.56 |
|  |  | TG C50:3 | 0.0019 | 0.008 | 0.58 | 2.23 ± 1.06 | 1.31 ± 0.62 |
|  |  | TG C50:4 | 0.009 | 0.024 | 0.59 | 0.65 ± 0.34 | 0.39 ± 0.18 |
|  |  | TG C50:5 | 0.0184 | 0.044 | 0.57 | 0.14 ± 0.08 | 0.08 ± 0.04 |
|  |  | TG C51:1 | 0.0005 | 0.003 | 0.6 | 0.34 ± 0.15 | 0.2 ± 0.07 |
|  |  | TG C51:2 | 0.0005 | 0.003 | 0.61 | 0.48 ± 0.19 | 0.29 ± 0.11 |
|  |  | TG C51:3 | 0.0034 | 0.011 | 0.66 | 0.28 ± 0.12 | 0.19 ± 0.07 |
|  |  | TG C52:1 | 0.0021 | 0.008 | 0.56 | 1.67 ± 0.98 | 0.94 ± 0.89 |
|  |  | TG C52:2 | 0.0028 | 0.01 | 0.68 | 9.1 ± 2.9 | 6.2 ± 2.6 |
|  |  | TG C54:1 | 0.0051 | 0.016 | 0.65 | 0.19 ± 0.09 | 0.12 ± 0.05 |
|  |  | TG C54:2 | 0.0034 | 0.011 | 0.67 | 1.31 ± 0.44 | 0.88 ± 0.37 |
|  |  | TG C55:2 | 0.0001 | 0.001 | 0.68 | 0.08 ± 0.02 | 0.05 ± 0.02 |
|  |  | TG C56:5 | 0.0113 | 0.03 | 0.75 | 0.47 ± 0.15 | 0.36 ± 0.16 |
|  |  | TG C58:5 | 0.0177 | 0.043 | 0.79 | 0.04 ± 0.01 | 0.03 ± 0.01 |
|  | ***Amines*** | | | | | | |
| Amines | | Kynurenine | 0.0008 | 0.007 | 0.69 | 0.008 ± 0.002 | 0.005 ± 0.001 |
|  |  | Saccharopine | < 0.0001 | < 0.001 | 0.55 | 0.006 ± 0.002 | 0.003 ± 0.001 |
|  |  | 4-Hydroxyproline | 0.0039 | 0.025 | 1.68 | 0.13 ± 0.05 | 0.23 ± 0.12 |
|  |  | Glutathione | 0.0009 | 0.007 | 0.72 | 0.003 ± 0.001 | 0.002 ± 0.001 |
|  |  | Pipecolic_acid | 0.0007 | 0.007 | 1.46 | 1.01 ± 0.23 | 1.48 ± 0.54 |
|  |  | s_Methylcysteine | 0.0074 | 0.04 | 1.70 | 0.2 ± 0.08 | 0.34 ± 0.19 |
|  |  | Sarcosine* | 0.0306 | 0.129 | 1.30 | 0.011 ± 0.004 | 0.014 ± 0.005 |
|  |  | Tryptophan* | 0.013 | 0.061 | 0.77 | 3.65 ± 0.91 | 2.83 ± 0.59 |
|  |  | γ-Glutamylglutamine | 0.0004 | 0.007 | 0.65 | 0.012 ± 0.004 | 0.007 ± 0.003 |
| Acyl Carnitines | | Betaine* | 0.0288 | 0.235 | 1.33 | 2.35 ± 0.98 | 3.13 ± 0.95 |
|  | ***Cholesterol esters*** | | | | | | |
|  | | CE C18:3 | 0.017 | 0.043 | 0.77 | 0.04 ± 0.01 | 0.03 ± 0.01 |
|  | ***Sphingomyelins*** | | | | | | |
| Positive Lipids | | SM C18:1/14:0 | < 0.0001 | < 0.001 | 0.70 | 0.45 ± 0.1 | 0.32 ± 0.07 |
|  |  | SM C18:1/21:0 | < 0.0001 | < 0.001 | 0.70 | 0.27 ± 0.05 | 0.19 ± 0.03 |
|  |  | SM C18:1/23:0 | 0.0001 | 0.001 | 0.78 | 0.67 ± 0.12 | 0.53 ± 0.08 |
|  |  | SM C18:1/25:0 | 0.0142 | 0.036 | 0.64 | 0.06 ± 0.02 | 0.04 ± 0.02 |
|  |  | SM C18:1/25:1 | 0.001 | 0.005 | 0.64 | 0.1 ± 0.03 | 0.07 ± 0.03 |
|  | ***Free fatty acid and Acyl-carnitines*** | | | | | | |
| Acyl Carnitines | | Nonanoylcarnitine | < 0.0001 | < 0.001 | 0.29 | 0.34 ± 0.14 | 0.1 ± 0.05 |
|  |  | Oleylcarnitine* | 0.0361 | 0.235 | 1.38 | 1.35 ± 0.44 | 1.87 ± 0.74 |
|  |  | Linoleylcarnitine | 0.001 | 0.013 | 1.79 | 0.65 ± 0.28 | 1.16 ± 0.57 |
| Negative Lipids | | FA C20:2 | 0.0103 | 0.023 | 2.21 | 0.24 ± 0.13 | 0.53 ± 0.38 |
|  |  | FA C18:2 | 0.0102 | 0.023 | 2.19 | 13.07 ± 8.16 | 28.57 ± 21.57 |
|  | ***Oxylipins*** | | | | | | |
| Oxylipins | | 5-HETrE | 0.0001 | 0.003 | 0.39 | 0.03 ± 0.02 | 0.01 ± 0.01 |
|  |  | 11-HDoHE* | 0.0037 | 0.065 | 2.68 | 0.02 ± 0.01 | 0.05 ± 0.04 |
|  |  | 14-HDoHE* | 0.0194 | 0.227 | 2.38 | 0.29 ± 0.31 | 0.68 ± 0.72 |
|  |  | 8,9-DiHETrE* | 0.0347 | 0.262 | 0.70 | 0.01 ± 0.003 | 0.004 ± 0.002 |
|  |  | 10-HDoHE* | 0.0442 | 0.262 | 1.76 | 0.04 ± 0.03 | 0.06 ± 0.05 |
|  |  | 12,13-DiHOME* | 0.045 | 0.262 | 0.53 | 3.65 ± 2.93 | 1.94 ± 1.50 |
| * q-value > 0.05 | | | | | | | |

***Table S2. Pattern analyses K-means serum metabolite scores and assigned clusters***

***(Additional File 3: Excel format)***

**Table S3. Descriptive statistics of significant serum metabolites between clinical phases**. The table contains their corresponding anova p value, pattern analyses cluster and phase descriptive statistics using normalised area ratios, ordered by metabolic class.

| Targeted Metabolomics Platform | Metabolite | p-value | Pattern analyse cluster | Healthy Controls Mean ± SD | IT Phase Mean ± SD | IA Phase Mean ± SD | IC Phase Mean ± SD | ENEG Phase Mean ± SD |
| --- | --- | --- | --- | --- | --- | --- | --- | --- |
|  |  |  |  |  |  |  |  |  |
| **Phospholipids** | | | | | | | | |
| Positive Lipids | PC C38:4 | 0.0432 | 15 | 3.94 ± 0.94 | 3.3 ± 0.59 | 3.15 ± 0.75 | 3.78 ± 0.95 | 3.95 ± 0.99 |
|  | PC C40:4 | 0.0478 | 14 | 0.08 ± 0.03 | 0.07 ± 0.03 | 0.06 ± 0.03 | 0.06 ± 0.03 | 0.09 ± 0.04 |
| **Lysophospholipids** | | | | | | | | |
| Negative Lipids | *sn1* LPC C20:3 (w3w6) | 0.0284 | 8 | 0.82 ± 0.33 | 0.44 ± 0.15 | 0.49 ± 0.16 | 0.47 ± 0.16 | 0.65 ± 0.25 |
|  | *sn2* LPC C20:3 (w3w6) | 0.0263 | 8 | 0.16 ± 0.06 | 0.09 ± 0.03 | 0.1 ± 0.03 | 0.09 ± 0.03 | 0.13 ± 0.05 |
|  | LPE C18:1 | 0.0490 | 15 | 0.07 ± 0.03 | 0.05 ± 0.02 | 0.06 ± 0.03 | 0.07 ± 0.03 | 0.07 ± 0.05 |
|  | LPE C20:3 (w3w6) | 0.0292 | 15 | 0.008 ± 0.003 | 0.005 ± 0.002 | 0.006 ± 0.003 | 0.007 ± 0.003 | 0.007 ± 0.003 |
|  | LPE C20:4 | 0.0226 | 15 | 0.079 ± 0.023 | 0.058 ± 0.015 | 0.057 ± 0.019 | 0.074 ± 0.021 | 0.073 ± 0.03 |
|  | *sn1* LPC C20:1 | 0.0323 | 21 | 0.047 ± 0.009 | 0.045 ± 0.008 | 0.061 ± 0.016 | 0.056 ± 0.017 | 0.054 ± 0.015 |
| **Triglycerides** | | | | | | | | |
| Positive Lipids | TG C54:2 | 0.0268 | 10 | 1.31 ± 0.44 | 0.88 ± 0.37 | 1.29 ± 0.78 | 1.23 ± 0.68 | 2.13 ± 2.14 |
|  | TG C54:3 | 0.0158 | 10 | 3.18 ± 0.83 | 2.64 ± 1.18 | 3.68 ± 1.84 | 3.28 ± 1.63 | 5.26 ± 3.8 |
|  | TG C54:4 | 0.0398 | 10 | 3.28 ± 1.33 | 3.35 ± 1.57 | 4.69 ± 2.09 | 3.59 ± 1.66 | 5.27 ± 3.41 |
|  | TG C55:3 | 0.0249 | 10 | 0.09 ± 0.02 | 0.08 ± 0.02 | 0.09 ± 0.03 | 0.09 ± 0.02 | 0.11 ± 0.03 |
|  | TG C56:3 | 0.0282 | 10 | 0.14 ± 0.04 | 0.12 ± 0.06 | 0.14 ± 0.07 | 0.14 ± 0.08 | 0.26 ± 0.29 |
|  | TG C56:4 | 0.0013 | 10 | 0.23 ± 0.06 | 0.19 ± 0.08 | 0.25 ± 0.09 | 0.22 ± 0.11 | 0.36 ± 0.25 |
|  | TG C56:5 | 0.0025 | 10 | 0.47 ± 0.15 | 0.36 ± 0.16 | 0.45 ± 0.23 | 0.41 ± 0.23 | 0.61 ± 0.26 |
|  | TG C56:6 | 0.0415 | 10 | 0.72 ± 0.28 | 0.6 ± 0.31 | 0.71 ± 0.34 | 0.65 ± 0.27 | 0.86 ± 0.34 |
|  | TG C58:5 | 0.0098 | 10 | 0.04 ± 0.01 | 0.03 ± 0.01 | 0.04 ± 0.01 | 0.04 ± 0.01 | 0.05 ± 0.03 |
|  | TG C42:1 | 0.0428 | 15 | 0.07 ± 0.05 | 0.03 ± 0.02 | 0.03 ± 0.01 | 0.06 ± 0.06 | 0.07 ± 0.09 |
|  | TG C52:2 | 0.0286 | 15 | 9.1 ± 2.87 | 6.24 ± 2.65 | 7.12 ± 2.89 | 7.55 ± 3.14 | 10.79 ± 5.36 |
|  | TG C55:2 | 0.0157 | 15 | 0.08 ± 0.02 | 0.05 ± 0.02 | 0.06 ± 0.03 | 0.06 ± 0.02 | 0.08 ± 0.03 |
| **Amines** | | | | | | | | |
| Amines | Ornithine | 0.0065 | 6 | 1.15 ± 0.27 | 1.37 ± 0.43 | 1.41 ± 0.29 | 1.47 ± 0.45 | 1.84 ± 0.45 |
|  | Phenylalanine | 0.0126 | 10 | 0.84 ± 0.16 | 0.83 ± 0.14 | 0.98 ± 0.1 | 0.97 ± 0.16 | 1 ± 0.2 |
|  | Glutamic acid | 0.0135 | 10 | 1.07 ± 0.38 | 1.14 ± 0.31 | 1.43 ± 0.42 | 1.37 ± 0.49 | 1.73 ± 0.66 |
|  | Methionine | 0.0392 | 10 | 0.015 ± 0.008 | 0.014 ± 0.014 | 0.013 ± 0.005 | 0.015 ± 0.005 | 0.015 ± 0.006 |
|  | Kynurenine | 0.0059 | 13 | 0.008 ± 0.002 | 0.005 ± 0.001 | 0.006 ± 0.002 | 0.006 ± 0.001 | 0.007 ± 0.002 |
|  | Citrulline | 0.0061 | 9 | 0.32 ± 0.06 | 0.31 ± 0.07 | 0.3 ± 0.08 | 0.33 ± 0.09 | 0.4 ± 0.08 |
| Acyl Carnitines | Carnitine | 0.0008 | 6 | 2.95 ± 0.87 | 3.26 ± 0.69 | 3.53 ± 0.73 | 3.19 ± 0.64 | 4.01 ± 0.64 |
|  | Choline | 0.0113 | 10 | 1.22 ± 0.26 | 1.37 ± 0.36 | 1.89 ± 0.54 | 1.56 ± 0.4 | 1.78 ± 0.43 |
| **Sphingomyelins** | | | | | | | | |
| Positive Lipids | SM C18:1/18:1 | 0.0112 | 12 | 0.35 ± 0.07 | 0.4 ± 0.05 | 0.33 ± 0.05 | 0.4 ± 0.06 | 0.37 ± 0.07 |
|  | SM C18:1/25:0 | 0.0346 | 15 | 0.06 ± 0.02 | 0.04 ± 0.02 | 0.04 ± 0.02 | 0.05 ± 0.02 | 0.05 ± 0.03 |
|  | SM C18:1/23:1 | 0.0023 | 7 | 0.52 ± 0.1 | 0.43 ± 0.05 | 0.4 ± 0.06 | 0.51 ± 0.08 | 0.45 ± 0.12 |
|  | SM C18:1/21:0 | 0.0231 | 7 | 0.27 ± 0.05 | 0.19 ± 0.03 | 0.2 ± 0.04 | 0.24 ± 0.05 | 0.23 ± 0.08 |
| **Free fatty acid and Acyl-carnitines** | | | | | | | | |
| Acyl Carnitines | Nonaylcarnitine | 0.0168 | 8 | 0.34 ± 0.14 | 0.1 ± 0.05 | 0.19 ± 0.12 | 0.16 ± 0.14 | 0.24 ± 0.18 |
|  | Isovalerylcarnitine | 0.0097 | 10 | 0.83 ± 0.49 | 0.68 ± 0.29 | 0.94 ± 0.41 | 0.78 ± 0.22 | 1.07 ± 0.47 |
|  | Stearoylcarnitine | 0.0295 | 10 | 0.47 ± 0.15 | 0.43 ± 0.13 | 0.5 ± 0.17 | 0.46 ± 0.13 | 0.58 ± 0.18 |
|  | 2-Methylbutyroylcarnitine | 0.0390 | 10 | 0.59 ± 0.21 | 0.49 ± 0.18 | 0.55 ± 0.18 | 0.53 ± 0.22 | 0.71 ± 0.29 |
|  | Decenoylcarnitine | 0.0226 | 18 | 1.25 ± 0.64 | 1.75 ± 1.19 | 1.74 ± 0.83 | 1.03 ± 0.58 | 1.41 ± 0.38 |
|  | Decanoylcarnitine | 0.0468 | 23 | 0.95 ± 0.81 | 1.02 ± 0.9 | 0.93 ± 0.5 | 0.6 ± 0.4 | 0.89 ± 0.42 |
| Negative Lipids | FA C20:4 (w6) | 0.0143 | 10 | 6.36 ± 4.91 | 6.31 ± 2.78 | 7.08 ± 2.82 | 5.93 ± 2.94 | 8.63 ± 3.09 |
|  | FA C20:3 (w3w6) | 0.0293 | 23 | 0.85 ± 0.31 | 0.9 ± 0.39 | 0.86 ± 0.33 | 0.75 ± 0.34 | 1.09 ± 0.36 |
|  | FA C22:4 | 0.0365 | 23 | 0.4 ± 0.16 | 0.49 ± 0.29 | 0.41 ± 0.19 | 0.38 ± 0.23 | 0.53 ± 0.17 |
| **Oxylipins** | | | | | | | | |
| Oxylipins | 12-HETE | 0.0472 | 14 | 8.63 ± 15.99 | 5.71 ± 7.43 | 6.4 ± 5.77 | 4.59 ± 2.96 | 11.19 ± 8.67 |
|  | 5,6-DiHETrE | 0.0486 | 21 | 0.017 ± 0.009 | 0.019 ± 0.014 | 0.037 ± 0.034 | 0.019 ± 0.014 | 0.033 ± 0.025 |
| **Diacylglycerol** | | | | | | | | |
| Positive Lipids | DG C36:2 | 0.0354 | 10 | 0.26 ± 0.07 | 0.22 ± 0.08 | 0.25 ± 0.09 | 0.24 ± 0.08 | 0.35 ± 0.17 |
